# Human umbilical cord mesenchymal stem cell-based gene therapy for hemophilia B using scAAV-DJ/8-LP1-hFIXco transduction

**DOI:** 10.1186/s13287-024-03824-y

**Published:** 2024-07-18

**Authors:** Zibin Bu, Jintu Lou, Weiqun Xu, Lingyan Zhang, Yongmin Tang

**Affiliations:** 1 Division/Center of Hematology-oncology, Children’s Hospital, Zhejiang University School of Medicine, National Clinical Research Center for Child Health, Zhejiang Hangzhou, 310003 PR China; 2 Department of Clinical Laboratory, Children’s Hospital, Zhejiang University School of Medicine, National Clinical Research Center for Child Health, Zhejiang Hangzhou, 310003 PR China

**Keywords:** Hemophilia B, Cell-based gene therapy, Human umbilical cord mesenchymal stem cells, scAAV-DJ/8-LP1-hFIXco

## Abstract

**Background:**

Hemophilia B is an X-linked bleeding disorder caused by a mutation in the gene responsible for encoding coagulation factor IX (FIX). Gene therapy offers promising potential for curing this disease. However, the current method of relatively high dosage of virus injection carries inherent risks. The purpose of this study was to introduce a novel scAAV-DJ/8-LP1-hFIXco vector transduced human umbilical cord blood derived mesenchymal stem cells (HUCMSCs) as an alternative cell-based gene therapy to conventional gene therapy for Hemophilia B.

**Methods:**

The LP1-hFIXco gene structure was designed by us through searching the literature from NCBI and the scAAV-DJ/8-LP1-hFIXco vector was constructed by a commercial company. The HUCMSCs were cultivated in routine approach and transduced with scAAV-DJ/8-LP1-hFIXco vector. The human FIX activation system was employed for detection of hFIXco activity. The RNA and protein expression levels of the hFIXco were evaluated using PCR and western blot techniques. In animal studies, both NSG and *F9-KO* mice were used for the experiment, in which clotting time was utilized as a parameter for bleeding assessment. The immunohistochemical analysis was used to assess the distribution of HUCMSCs in mouse tissue sections. The safety for tumorigenicity of this cell-based gene therapy was evaluated by pathological observation after hematoxylin-eosin staining.

**Results:**

The transduction of HUCMSCs with the scAAV-DJ/8-LP1-hFIXco vector results in consistent and sustainable secretion of human FIXco during 5 months period both in vitro and in mouse model. The secretion level (hFIXco activity: 97.1 ± 2.3% at day 7 to 48.8 ± 4.5% at 5 months) was comparable to that observed following intravenous injection with a high dose of the viral vector (hFIXco activity: 95.2 ± 2.2% to 40.8 ± 4.3%). After a 5-month observation period, no clonal expansions of the transduced cells in tissues were observed in any of the mice studied.

**Conclusions:**

We have discovered a novel and safer HUCMSCs mediated approach potentially effective for gene therapy in hemophilia B.

**Supplementary Information:**

The online version contains supplementary material available at 10.1186/s13287-024-03824-y.

## Background

Hemophilia B is an X-linked bleeding disorder resulting from a mutation in the gene responsible for encoding coagulation factor IX (FIX). Gene therapy utilizing an adenovirus-associated virus (AAV) vector has been shown significant potential for long-term treatment of hemophilia B with just one injection of the vector. In November 2022, the U.S. Food and Drug Administration (FDA) approved the first gene therapy medicine Hemgenix for the treatment of adult patients with hemophilia B, using AAV5 as the vector to deliver the FIX variant padua [1]. However, the efficient transduction of AAV is hindered by the requirement to convert its single-stranded (ss) genome into double-stranded (ds) forms that can be transcribed in target cells. Despite our inability to find evidence supporting Padua’s efficiency, according to Halder SK et al. [2], protein variants are most likely to be deleterious to protein structure or function. The genuine effectiveness of Padua in humans remains to be confirmed. CSL Behring, an Australian drug manufacturer, has priced a single dose of Hemgenix at US $3.5 million, thereby establishing it as the most costly drug globally. Its exorbitant cost [3] may limit its clinical application.

AAV serotypes exhibit significant differences in transduction efficiencies and tissue tropisms, making them highly promising vectors for human gene therapy. However, in practice, their application in patients is limited by the prevalence of anti-AAV immunity or due to inadequate performance in specific targets. AAV-DJ is a chimera of adenovirus-associated virus types 2, 8, and 9, while AAV-DJ/8 is a mutant of AAV-DJ in heparin binding domain (HBD). AAV-DJ/8 differs from its closest natural relative (AAV-2) by 60 capsid amino acids. In culture, recombinant AAV-DJ/8 vectors outperformed eight standard AAV serotypes, and in livers, they greatly surpassed AAV-2 [4].

With low immunogenicity in humans [5, 6], high transduction efficiency in liver cells [7, 8], and high factor IX (FIX) production, we have developed a novel self-complementary recombinant adeno-associated virus vector (scAAV-DJ) with HBD mutation (scAAV-DJ/8) that expresses human codon-optimized factor IX (scAAV-DJ/8-LP1-hFIXco). This vector shows promise as a potential candidate for gene therapy for hemophilia B, which may mainly exist in target cells as episomes in the form of circular genomes or concatemers [9].

In the field of gene therapy, safety for recipients is always a primary concern in research, encompassing concerns such as organ toxicity, immune responses, and tumorigenicity. Numerous studies have consistently shown that recombinant adeno-associated virus (rAAV) exhibits a favorable safety profile with minimal toxicity in various cells and tissues [10–12]. However, A recent case report revealed that a patient with advanced Duchenne’s muscular dystrophy (DMD) treated with high-dose rAAV9 (1 × 10^14^ vector genomes (vg)/kilogram（kg)) gene therapy died from an innate immune reaction caused acute respiratory distress syndrome (ARDS) [13]. Although the dosage of the current intravenous injection of the vector with approximately 10^11^-10^12^ vg/kg necessary for a therapeutic response is 100–1000 fold lower than that in the DMD case report, it may still pose the potential risks of liver toxicity and immune reactions. In animal models, high vector doses are observed to be associated with non-specific biodistribution of the vectors [14]. Moreover, the production of clinical-grade vectors at a dosage of 10^11^-10^12^ vg /Kg per patient can be economically burdensome [3].

Giang N. Nguyen et al. [15] revealed AAV gene therapy in dogs with hemophilia A identifies clonal expansions of transduced liver cells. They identified a residential AAV vector including a whole FVIII gene integrated in gene DLEU2 (deleted in leukemia 2) in one dog. The integration of the host genome has been extensively studied in hemophilia B gene therapy research [16–18], demonstrating its safety through numerous experiments. Random integration into the host genome may indeed occur [17]. The concern regarding tumorigenicity associated with gene therapy remains to be a persistent issue.

In this study, we introduced the human umbilical cord mesenchymal stem cells (HUCMSCs) for cell based gene therapy in hemophilia B. HUCMSCs are stromal cells that possess the capacity for self-renewal and demonstrate differentiation into multiple cell lineages [19, 20]. Furthermore, they exhibit low immunogenicity and have immunomodulatory effects which make them suitable for targeted cell-based therapies [21]. Safety of commercially sourced HUCMSC infusions has been established in clinical trials with patients. Its clinical application for preventing and treating graft-versus-host disease (GVHD) or stimulation of hematopoiesis recovery after hematopoietic stem cell transplantation is currently underway [22].

Given the aforementioned points, every approach possesses both advantages and disadvantages in terms of effectiveness, toxicity, tumorigenicity, etc. Maintaining effectiveness while minimizing side effects remains a crucial issue. We hypothesize that transducing host cells in vitro prior to infusion with reduced doses of vectors may offer a solution to this challenge.

In this study, instead of directly injecting a high dose of vector into the tail vein of the mouse, we have discovered that HUCMSCs transduced by a novel scAAV-DJ/8-LP1-hFIXco vector (DJ/8-hFIX) could be employed in a specific cell-based gene therapy approach for hemophilia B. This alternative method has the potential to mitigate the risks associated with high-dose vector injection, thereby avoiding direct exposure to AAV vectors and associated risks. Additionally, it could pave a path for AAV-mediated cell-based gene therapy in hemophilia B.

## Materials and methods

### Materials

The refined human FIX gene sequence was designed by our team. Then the gene sequence was sent to GeneChem Biotechnology Company (Shanghai, China) to integrate into scAAV vector. The AAV-DJ/8 Helper Free Bicistronic expression system (IRES-GFP) kit and QuickTiterTM AAV Quantitation kit were purchased from Cell Biolabs (San Diego, USA). The human embryonic kidney 293T cell line (HEK293T), Chinese hamster ovary cell line (CHO), normal human liver cell line (HL7702), and human amnion cell line (FL) were acquired from ATCC and cryopreserved in the Hematology-Oncology Laboratory at the Division of Hematology-Oncology, Children’s Hospital, Zhejiang University School of Medicine. Treatment grade HUCMSCs were purchased from Qilu Cell Therapy Engineering Technology Co. Ltd (Shandong, China). Mouse anti-human FIX monoclonal antibody was purchased from Abcam (#ab17196; Billerica, MA). Mouse anti-human CD105 antibody was purchased from proteintech (10862-1-AP; PTG, USA). Basal medium for HUCMSCs supplemented with 10% fetal bovine serum (FBS, Gibco), mesenchymal stem cell growth factor, and 1% penicillin/streptomycin (Invitrogen) was purchased from Viraltherapy Technologies (Wuhan, China). HyQ RPMI1640 (improved type) cell growth medium containing 15% FBS for HEK293T, CHO, HL7702, and FL cell lines was purchased from Hyclone (Logan, USA). polyethylene glycol (PEG) was purchased from Sigma-Aldrich (USA). Native purified human FXI protein was purchased from Abcam (#ab62538, Billerica, MA). The anticoagulant medicine Bax 326 was purchased from Abmole Bioscience Inc (Houston, USA). High Sieving Agarose was purchased from Yeasen Biotechnology Co.Ltd (Shanghai, China). The animal anesthetic tribromoethanol was purchased from Nanjing AIBI Bio-Technology. co. Ltd (Nanjing, China).

## Methods

### The construction of scAAV-LP1-hFIXco vector

**Fig. 1:**
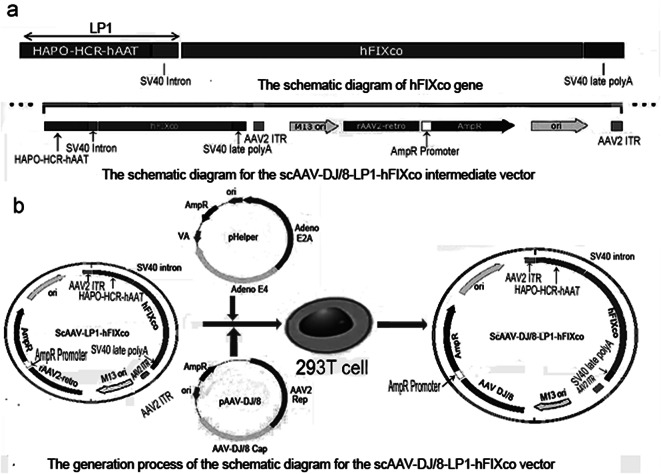
Gene structure of the designed hFIXco and schematic diagram illustrating the construction of DJ/8-hFIX vector. (**a**) Gene structure map of hFIXco carrying HAPO-HCR-hAAT promoter; hFIXco carrying HAPO-HCR-hAAT promoter is packaged into scAAV to generate scAAV-LP1-hFIXco. (**b**) The DJ/8-hFIX vector was successfully generated using scAAV-LP1-hFIXco, pAAV-DJ/8, and pHelper in 293T cells

The LP1 promoter was constructed with consecutive segments of the human apolipo-protein (HAPO) hepatic control region (HCR) and the human alpha-1-antitrypsin (hAAT) gene promoter including the 5’ untranslated region, which was cloned upstream of a modified SV40 small t antigen intron (SV40) [23, 24]. A codon-optimized human FIX (hFIXco) was designed by codons, which were most frequently found in highly expressed eukaryotic genes [25], synthesized by oligonucleotides, and subsequently assembled by ligation, PCR amplified, and sequenced prior to cloning into the scAAV backbone to create scAAV-LP1-hFIXco vector (Fig. 1a). The synthesis, identification of scAAV-LP1-hFIXco was performed by GeneChem Biotechnology Company (Shanghai, China).

### Construction and amplification and the titer detection of DJ/8-hFIX

The HEK293T cell line was used for DJ/8-hFIX packaging and amplification [26]. Three vectors including scAAV-LP1-hFIXco, pAAV-DJ/8 and pHelper for packaging was prepared at 1:1:1 ratio (F1b). The amplification process of DJ/8-hFIX was carried out in accordance with the instructions provided by the AAV-DJ/8 Helper Free Bicistronic Expression System (IRES-GFP) kit. The titer of DJ/8-hFIX was determined using the Quick Titer TM AAV Quantification kit as per the provided instructions.

### Cell culture

The HUCMSCs, HEK293T, CHO, FL and HL7702 cells were cultured in a humidified incubator at 37℃ with a 5% CO2 atmosphere. The cells were seeded in T75 flasks and used for experiment when they reached 80–95% of confluence.

### Activated partial thromboplastin time (APTT) assay and the activity assay of hFIXco

The APTT assay, a one-stage clotting factor assay, was conducted in the human factor IX deficient plasma by employing a phospholipid surface activator (made from rabbit brain powder) under the working concentration of CaCl_2_ (0.025 mol/L). The human factor IX deficient plasma was provided by Siemens (Germany). The analysis was performed using a fully automated coagulation analyzer (CS-5100 system, Sysmex Corporation, Japan). FIX: C was estimated using a reference curve prepared from standard human plasma (Siemens Healthcare GmbH, Germany), which was calibrated by the manufacturer against the WHO international standard for FIX. The calibrator signed the FIX percent was also provided by Siemens (Germany). A given percentage of FIX activity such as 100%, 80% etc. can be found on the standard curve.

Samples from cell supernants, NSG mice and F9-Knock out (*F9-KO*) mice centrifuged at 5000 g for 5 min before test.

In NSG mice study, the blank group’s APTT, which was injected with 0.9% Normal Saline (0.9% NS) was calibrated as zero when the results shows(in normal NSG mice(*n* = 4), the FIX assay was 57.8%±2.3% by our system, and all the NSG mice’s FIX activity data presented was substracted by this).

The use of a hemophilia B patient serum sample was approved by institutional ethnic review board of Children’s Hospital under approval number 2015-IRB-Q.5. The sample with a FIX activity of 1.7% and APTT of 62.4 s (the normal range for APTT is 23–38 s) was utilized. Employing the supernatant of tr-HL7702 as the positive control, the tr-HUCMSCs supernatant was combined with the hemophilia B patient’s serum at varying ratios: 1:8, 1:4, 1:2, 1:1, and 2:1.

### The transduction of host cells by DJ/8-hFIX vector

HUCMSCs, CHO, HL-7702, and FL cells were co-incubated with DJ/8-hFIX at a optimized ratio of 1:1000 after serial dosing experiment. Cell supernatants were collected at 24, 48, and 72 h, and hFIXco activity was assessed using an automatic coagulation analyzer. The DJ/8-hFIX transduced HL-7702 cells (tr-HL7702) served as the positive control, while the DJ/8-hFIX transduced CHO (tr-CHO) and FL cell lines (tr-FL) were employed as negative controls.

### RNA assay

Total mRNA was extracted using the Trizol method. Amplification of the hFIXco gene was performed using RT-PCR in the transduced cells. The forward and reverse primers for hFIXco were 5’-TACAACTCTGCAAGCTGGA-3’ and 5’-GTTCTTGCTCAAAGCCAA-3’, respectively. The size of the product was 243 bp. GAPDH served as the housekeeping gene. The forward and reverse primers for GAPDH amplification were 5’-TTCACCACCATGGAGAAGGC-3’ and 5’-GGCATGGACTGTGGTCATGA-3’, respectively. The size of the product was 192 bp.

### Western blot analysis

Pre-treated with the 0.2mL M-PER protein extraction buffer, the DJ/8-hFIX transduced HUCMSCs (tr-HUCMSCs, 10^7^) and its supernatants (from 24 h to 5 months) were mixed with sodium dodecyl sulphate polyacrylamide gel electrophoresis (SDS-PAGE) sample buffer. The DJ/8-hFIX untransduced HUCMSCs (untr*-*HUCMSCs) and their supernatants served as negative control. A positive control was established using pure human FIX protein (8ng/lane). Subsequently, the samples underwent resolution on an SDS-Tris-glycine acrylamide gel and were transferred onto a nitrocellulose membrane. Immunoblotting was conducted using the anti-hFIX monoclonal antibody (mouse anti-human), followed by incubation with the Goat anti-mouse horseradish peroxidase (HRP)-conjugated antibody and SuperSignal chemiluminescence substrate, respectively. The bands were visualized by exposing the x-ray film.

### Animal procedures

NOD-SCID gamma mice (NSG), (NOD.Cg-Prkd^cscid^ IL2rgtm^1Wjl^/SzJ ) mice and *F9*-Knock out (*F9-KO*) mice (strain name: C57BL/6JSmoc-*F9*^*em1Smoc*^) were obtained from the Shanghai Model Organisms Center, Inc (Shanghai, China) and kept in specific pathogen-free conditions. All experimental procedures involving animals followed institutional guidelines and were approved by the Institutional Animal Care and Use Committee (IACUC), Zhejiang Center of Laboratory Animals (ZJCLA). The mice used in the study were all male, 4 weeks old, and weighed approximately 20 gram (g). For the experimental groups, NSG mice and *F9-*Knock out mice were injected with tr-HUCMSCs (1 × 10^3^/g) via tail vein. Another group of mice received untr-HUCMSCs (1 × 10^3^/g) and 0.9% Normal Saline (0.9% NS) as a negative control and blank, respectively. The DJ/8-hFIX (1 × 10^11^vg/kg) tail vein injection group served as the positive control. Each NSG and *F9-KO* group consisted of four animals. Blood samples were collected at designated time points post-injection from the retroorbital venous plexus of NSG mice and the tail veins of *F9-KO* mice. Citrated plasma was centrifuged at 5000 g for 5 min, and the supernatant was stored at -80℃ for further assays.

### Human FIX activity assessment in *F9-KO* mice

Tribromoethanol was used to anesthetize the *F9-KO* mice. To obtain blood spots, we transected the tail of each experimental mouse at a length of 1 cm using a scalpel. Subsequently, blood spots were imaged using a SONY 5600 W camera, captured 30 s (s) post-incision. At the end of the 30-second period, we gently touched the wound site on the tail with filter paper at regular intervals until the blood spots disappeared. The clotting time was then recorded.

### Immunohistochemical analysis and hematoxylin-eosin staining

After the 7-day and 5-month post-transplantation periods, tissues including lung, liver, brain, bone marrow, and spleen were collected from various groups following carbon dioxide-induced euthanasia. The samples were fixed in 10% formalin and then embedded in paraffin wax. The paraffin sections were then deparaffinized, followed by antigen retrieval via microwave heating (at 500 W, 5 min x 2) in a pH 6.0 citrate buffer. The endogenous enzymes in the sections were blocked using 3% H_2_O_2_ for 20 min. Then, the sections were incubated overnight at 4 °C with the primary antibody (anti-human CD105 antibody, the specific membrane antigen of HUCMSCs), followed by HRP-conjugated goat anti-rabbit immunoglobulin G and then immersed in Diaminobenzidine chromogen substrate for 10 min. After being washed with distilled water and stained with Mayer hematoxylin, the sections were dehydrated and mounted with a coverslip. For the negative control, PBS (phosphate buffer saline) was substituted for the primary antibody. Tissues from 0.9% NS treated NSG mice incubated with anti-human CD105 antibody were also employed as a negative control. Immunohistochemical analysis, Hematoxylin-eosin staining was conducted routinely. Software used for image processing is 3DHISTECH’s Slide Converter. The 40x magnification of immunohistochemical photos was taken and used in this article.

The work has been reported in line with the ARRIVE guidelines 2.0.

### Statistical analysis

The statistical analysis was conducted using SPSS-13.0 software from Chicago, USA. The numerical data was presented as mean ± standard deviation. The paired Student’s t-test was utilized to assess significant differences between various treatments. A p-value below 0.05 was considered to be statistically significant.

## Results

### AAV-DJ/8 construction and transductions of the HUCMSCs

Using the AAV-DJ/8 Helper Free Bicistronic expression system (IRES-GFP) kit, DJ/8-hIX was successfully constructed and efficiently amplified in HEK293T cells with a vector ratio of 1:1:1 (consisting of scAAV-LP1-hFIXco, pAAV-DJ/8, and pHelper). The PEG concentration method was employed to achieve a high titer of DJ/8-hFIX. The titration of the DJ/8-hFIX was performed according to the instructions of the QuickTiterTM AAV Quantitation Kit.

HUCMSCs, CHO, HL-7702, and FL cells were incubated with scAAV-HP1-DJ/8-hFIXco vector at a concentration of 1:1000, before which we evaluated the ratios of 1:1000, 1:10000 and 1:100000, and found that the activity assay of hFIX secreted by HUCMSCs and HL-7702 remained relatively constant across all three concentrations. Eventually, we identified an optimal ratio of 1:1000 as the condition for further experiment. The DJ/8-hFIX demonstrated effective transduction in HUCMSCs and HL7702 cell lines. Notably, CHO and FL cell lines exhibited lower transduction efficiency.

### The activity evaluation of the hFIXco in the supernatants from the transduced cells

**Fig. 2:**
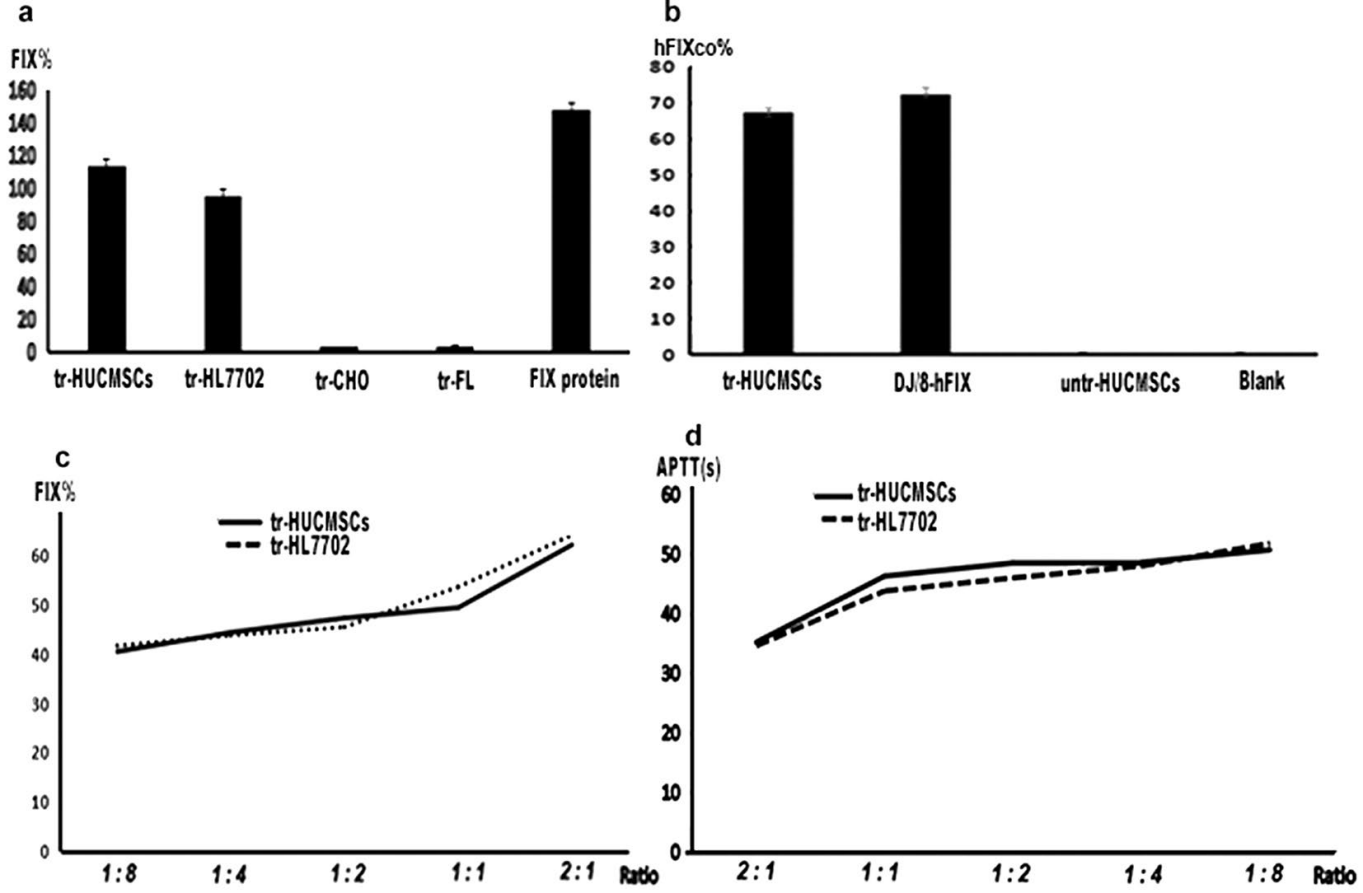
hFIXco secretion in different transduced cell types, *F9-KO* mice and its activity in serum of Hemophilia B patient. (**a**) After transducing HUCMSCs, HL7702, CHO, and FL cell lines with DJ/8-hIX for 24 h, we detected the activity of hFIXco in the supernatants. For comparison, a pure hFIX protein sample was used as a positive control (*n* = 4). (**b**) the activity assessment of hFIXco in *F9-KO* mice after 60 days’ injection with tr-HUCMSCs and DJ/8-hFIX (*n*= 4 per group). (1) tr-HUCMSCs (1 × 10^3^ cells/g) injection. (2) DJ/8-hIX(1 × 10^11^ vg/kg) injection. (3) untr-HUCMSCs (1 × 10^3^ cells/g) injection. 4.Blank (*F9-KO* mice with 0.9% NS injection). (**c**) The assessment of the activity of hFIXco produced by the tr-HUCMSCs and tr-HL7702 in the blood serum of a Hemophilia B patient (*n* = 1). (**d**) APTT improvement in Hemophilia B patient was observed after the addition of the supernatant derived from tr-HUCMSCs and tr-HL7702 (*n* = 1)

We used the human FIX activation system to evaluate the activity of the hFIXco secreted from the cells. After 24 h of transduction, the activity assay revealed that the hFIXco secreted by the tr-HUCMSCs was 113% ±4.92% compared to that of HL7702 (95.56%±1.29%). By contrast, the tr-CHO and tr-FL only exhibited a detection rate of active hFIXco at 2.52 ± 0.08% and 3.66 ± 0.12%, respectively (Fig. 2a).

To evaluate the authentic biological function of hFIXco secreted by tr-HUCMSCs, a serum sample from a hemophilia B patient with a FIX activity of 1.7% and APTT of 62.4 s (the normal range of APTT is 23–38 s) was utilized. Employing the supernatant of tr-HL7702 as the positive control, the tr-HUCMSCs supernatant was combined with the hemophilia B patient’s serum at varying ratios: 1:8, 1:4, 1:2, 1:1, and 2:1. Subsequently, the APTT was reduced to 50.6, 48.5, 48.4, 46.2, and 35.1 s, whereas the FIX activity increased to 40.4%, 44.3%, 47.2%, 49.3%, and 62% respectively. When compared to tr-HL7702 (APTT values of 51.7, 47.9, 45.9, 43.7, and 34.5 s, FIX activities of 41.6%, 43.7%, 45.4%, 53.5%, and 64% respectively), the hFIXco secreted by tr-HUCMSCs displayed coagulation capabilities comparable to those of HL7702. Both were capable of elevating the patient’s FIX activity to approximately 40% (as shown in Fig. 2c, d).

**Fig. 3:**
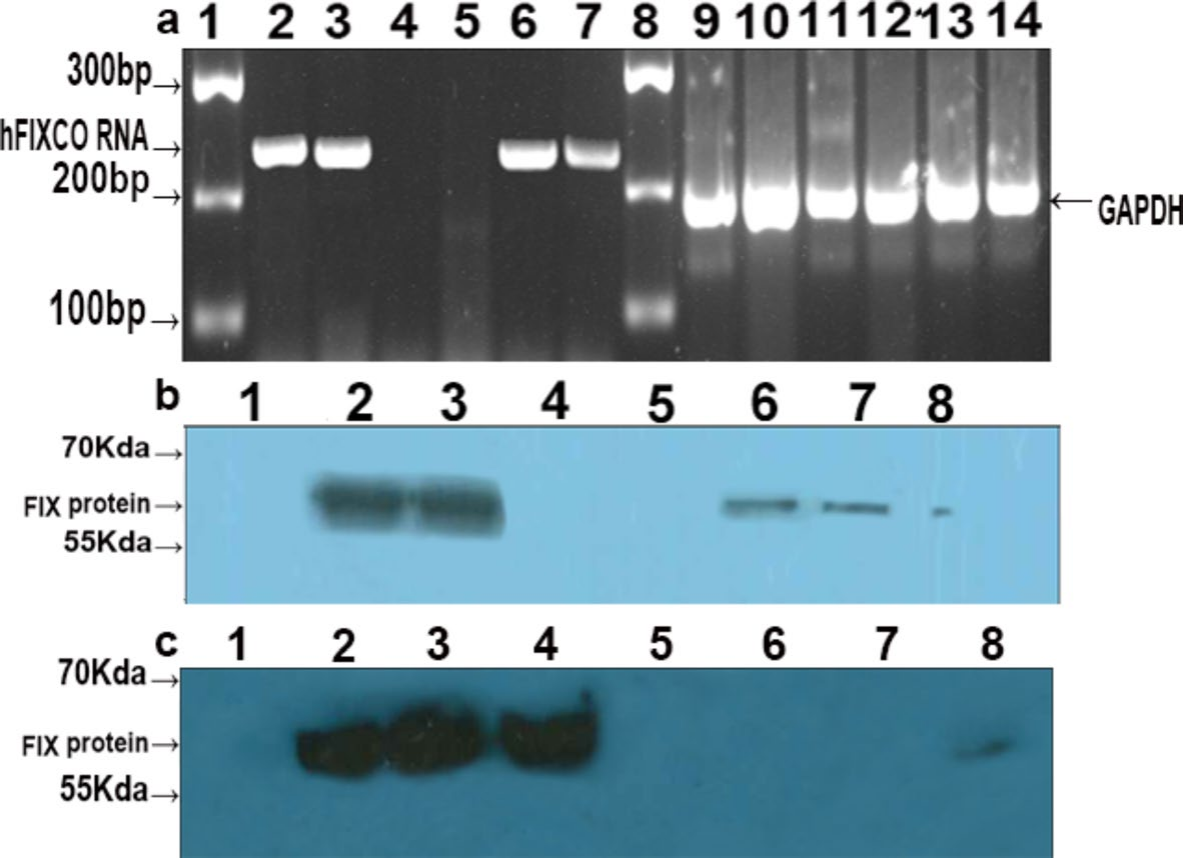
The expression of hFIXco in tr-HUCMSCs and tr-HL7702 by PCR and Western blot. (**a**) hFIXco RNA was detected in tr-HUCMSCs and HL7702 using RNA detection techniques (the figure presented is the cropped gel. Full-length gels are presented in Supplementary Material 4). The gel lanes were labeled as follows: Lane 1: marker, Lane 2 and Lane 3: tr-HUCMSCs cultured for 24 h and 5 months respectively, Lane 4 and Lane 5: untr-HUCMSCs cultured for 24 h and 5 months, respectively, Lane 6 and Lane 7: tr-HL7702 cultured for 24 h and 5 months respectively. Lane 8: marker. Lanes 9–14: GAPDH served as an internal control in the above-mentioned samples. (**b**) the FIX protein detection in the supernatants of tr-HL7702 (the figure presented is the cropped blot. The full-length blot is presented in Supplementary Material 5). The samples were loaded as follows: Lane 1: marker, Lane 2 and Lane 3: supernatant of tr-HL7702 after 24 h and 5 months culture, Lane 4 and Lane 5: supernatant of tr-CHO after 24 h and 5 months culture, Lane 6 and Lane 7: supernatant of HL7702 after 24 h and 5 months culture, Lane 8: pure FIX protein (8ng/lane). (**c**) the hFIXco protein detection in the supernatants and cell lysates of tr-HUCMSCs (the figure presented is the cropped blot. Full-length blot is presented in Supplementary Material 7). The samples were loaded as follows: Lane 1: marker, Lane 2 and Lane 3:supernatant of tr-HUCMSCs after 24 h and 5 months culture, Lane 4: cell lysate of tr-HUCMSCs after 5 months of transduction, Lane 5 and Lane 6: supernatant and cell lysate of untr-HUCMSCs for 24 h, Lane 7: blank medium. Lane 8: pure FIX protein (8ng/lane)

We used PCR to continuously monitor the RNA levels of hFIXco from the initial time point (24 h) until the final time point (five months). The RNA expression levels in the tr-HUCMSCs were similar to those observed in the HL7702 cell line (Fig. 3a). The hFIXco RNA expression band displayed a notable difference compared to the non-transduced samples.

Western blot analysis was used to detect the expression of the FIX protein in HL7702 and tr-HL7702 cells. Notably, in tr-HL7702 cells, the FIX protein expression was significantly higher compared to that in normal HL7702 cells (Fig. 3b). However, in normal HL7702 cells, weak bands indicative of FIX protein expression were observed. The western blot analysis was also employed to assess the expression of hFIXco protein in both tr-HUCMSCs and their supernatants. Our observations indicate that the level of hFIXco protein in the supernatant secreted by tr-HUCMSCs following 5 months of continuous culture was comparable to that observed at 24 h post-transplantation (Fig. 3c).

**Fig. 4:**
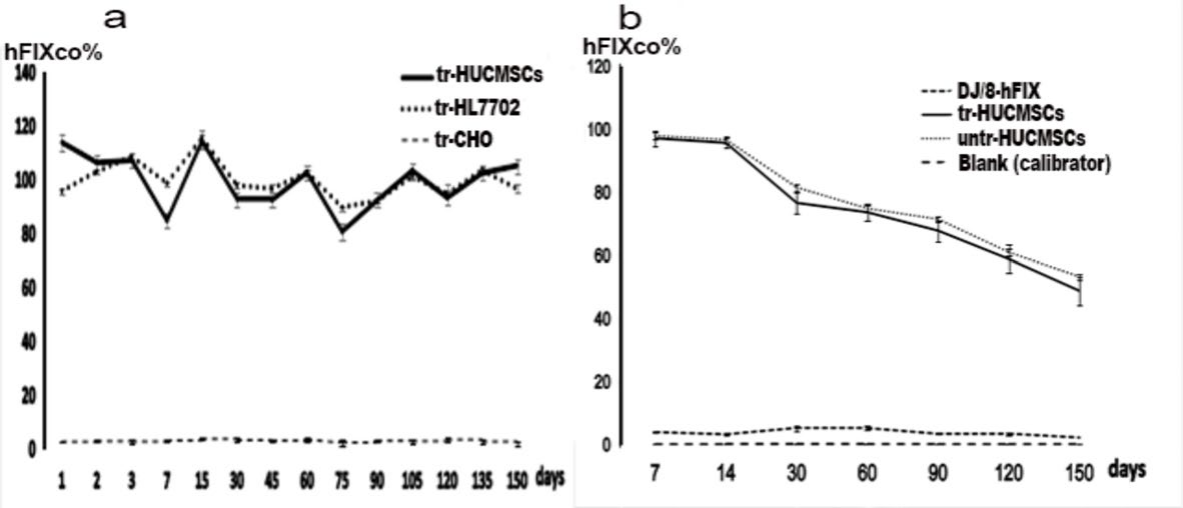
The stability and continuity of hFIXco secretion in tr-HUCMSCs in vitro and in vivo. (**a**) From 24 h to five months, The stability and continuity of hFIXco activity secreted by the tr-HUCMSCs, tr-HL7702, and tr-CHO cells were observed (*n* = 4, *P* < 0.05). (**b**) From 7 days to 5 months, using the blank (0.9% NS injection) group as calibrated as zero, the activity of hFIXco remained consistent gradually decreased in the NSG mice injected with tr-HUCMSCs and directly with DJ/8-hFIX (*n* = 4, *P* < 0.05). Notably, mice injected with untr-HUCMSCs exhibited minimal hFIXco activity

hFIXco activity was detected in the supernatants of tr-HUCMSCs from the first day post-transplantation to 5 months of cultivation, with levels ranging from approximately 113%±4.92% to around 105%±1.13%, which was consistent with that of tr-HL7702, When compared to tr-CHO, a significant difference was observed (Fig. 4a).

### The activity and coagulation function assay of the hFIXco in mouse model

In our study, we assessed the secretion of hFIX protein from tr-HUCMSCs in NSG mice over the course of 7 days to 5 months. The activity of hFIX protein remained stable and gradually decreased in both the tr-HUCMSCs and DJ/8-hFIX injection groups. In the tr-HUCMSCs group, hFIXco activity decreased from 97.1 ± 2.3% at day 7 to 48.8 ± 4.5% at 5 months. Similarly, in the DJ/8-hFIX injection group, hFIXco activity decreased from 95.2 ± 2.2% to 40.8 ± 4.3%. However, almost no hFIXco activity was observed in the groups of mice injected with untr*-*HUCMSCs or 0.9% NS. These findings suggest that the hFIXco activities derived from tr-HUCMSCs exhibit a pattern of stability and gradual decline similar to that observed in the DJ/8-hFIX direct injection group (See Fig. 4b).

In the *F9-KO* study, the activity of hFIXco in sera was measured 60 days after injection using an activity assay. The tr-HUCMSCs group displayed an average hFIXco level of 67.4 ± 1.3%. This result was significantly higher than that of the untr-HUCMSCs group (0.3%±0.1%, *P* < 0.05) and the blank group (0.2%±0.1%, *P* < 0.05). Nevertheless, there was no significant difference in comparison to the DJ/8-hFIX direct injection group, which exhibited an average hFIXco level of 72.6%±1.7% (Fig. 2b).

**Fig. 5:**
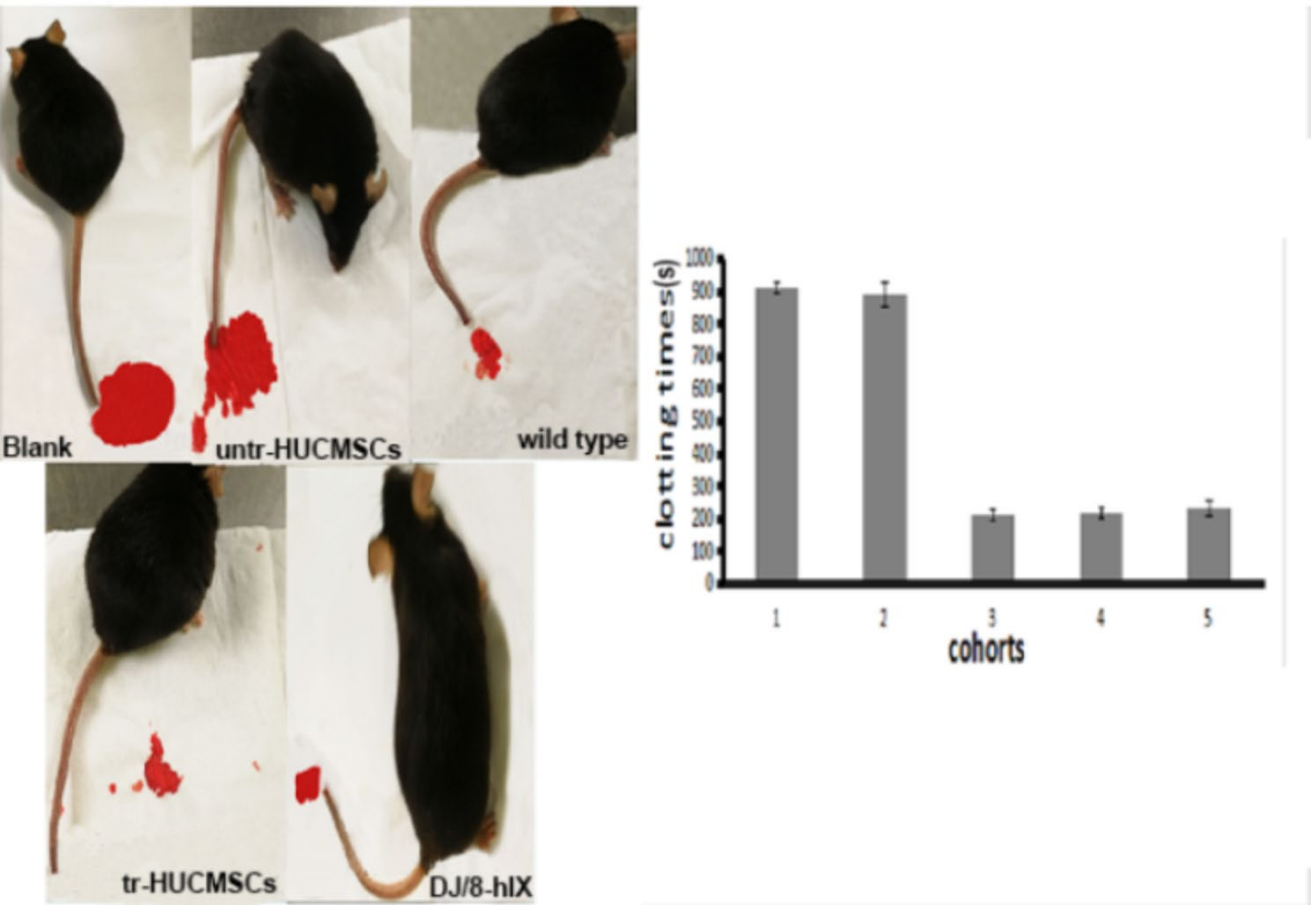
Clotting time of all the experiment cohorts at 90 days after transplantation (*n* = 4 per group). (1) Blank (*F9-KO* mice with 0.9% NS injection). (2) untr*-*HUCMSCs (1 × 10^3^ cells/g) injection. (3) wild type (same strain as *F9-KO* mice, but without the *F9* gene knocked out). (4) tr-HUCMSCs(1 × 10^3^ cells/g) injection. 5.DJ/8-hIX (1 × 10^11^ vg/kg) injection. The *F9-KO* mice were anesthetized using tribromoethanol. The tail was cut by 1 cm, and photographs were taken 30 s post-incision. For test Clotting time, the filter paper was used to touch the wound at regular intervals until blood cessation was observed

To assess whether the hFIXco protein produced by exogenous transgenes has a physiological activity that improves clotting, the tails of *F9-KO* mice were surgically amputated at a length of 1 cm to measure their clotting times. At 90 days post-injection, the clotting times of mice in the DJ/8-hFIX direct injection group and the tr-HUCMSCs group were similar to those of wild-type mice (less than 240 s), and significantly shorter than those of mice in the untr-HUCMSCs group and the group injected with 0.9% NS. In mice from both the untr-HUCMSCs group and the group injected with 0.9% NS, the bleeding time exceeded 800 s and stopped bleeding with Bax 326 eventually. Mice that were directly injected with DJ/8-hFIX or tr-HUCMSCs survived despite experiencing minor bleeding for approximately 200 s. The results suggest that the injection of pure DJ/8-hFIX (1 × 10^11^ vg/kg) or transplantation of tr-HUCMSCs (1 × 10^3^ cells/g) can exert therapeutic effects as observed by the shortened bleeding time (Fig. 5).

### Safety assessment of cell-based gene therapy in NSG mice

**Fig. 6:**
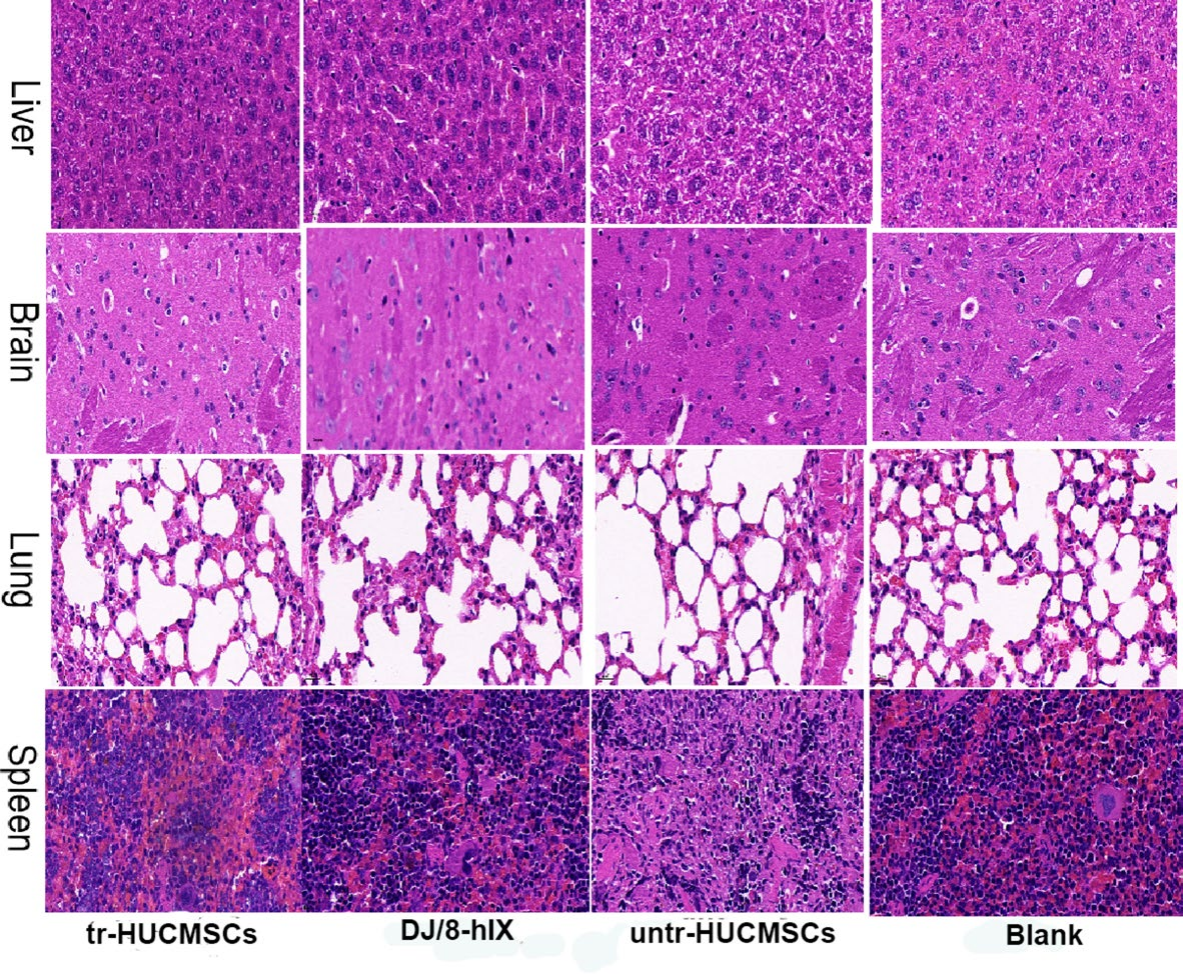
Hematoxylin-eosin stain in the tissue of NSG mice after 5 months of transplantation. Animal tissues such as liver, spleen, lung and brain were collected for hematoxylin-eosin stain (40×)

To elucidate the possible concerns regarding tumorigenicity induced by gene therapy, we conducted an experiment with NSG mice. The mice, which was depicted as immune deficiency and easy to tumorigenicity, seemed healthy during the whole experiment. Tissues such as brain, lungs, liver, and spleen from NSG mice were collected at the end of experiment (at 5 months post-transplantation). Furthermore, The remaining mice continued to survive despite an additional 2-month extension of the experiments. Hematoxylin-eosin staining of tissues revealed no significant differences among all groups. No clonal expansion of transduced cells was observed in either group after 5 months of experimentation (Fig. 6).

### Evaluation of the cell distribution in NSG mice

**Fig. 7:**
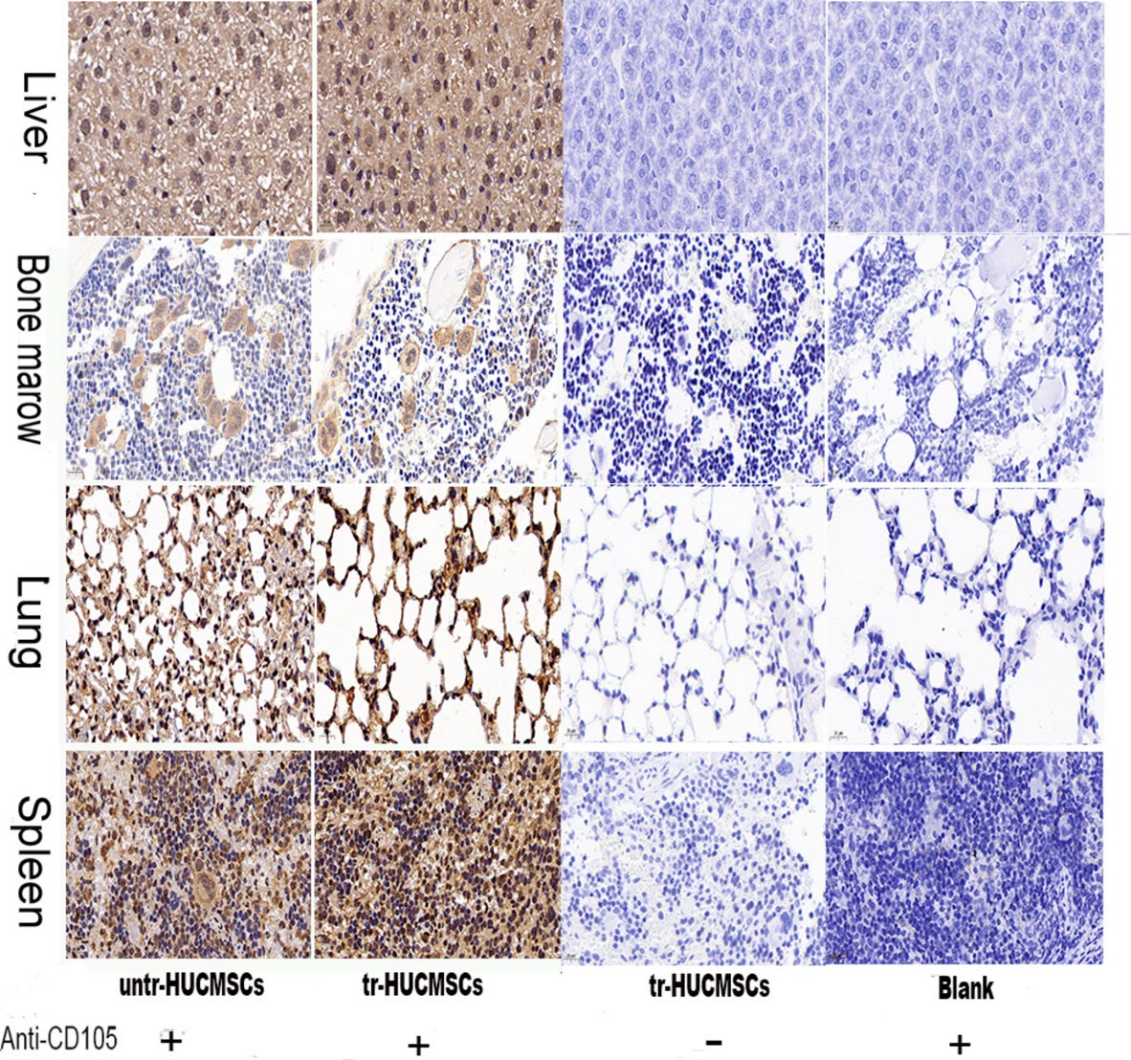
Immunohistochemical analysis of the distribution of tr-HUCMSCs or untr-HUCMSCs in NSG mice after 2 weeks transplantation. Selected animal tissues such as liver, spleen, lung and bone marrow were collected for immunohistochemical analysis (40×). ( Blank: injected with 0.9% NS. Anti-CD105 +: adding anti-CD105 antibody; anti-CD105 -: no adding anti-CD105 antibody )

**Fig. 8:**
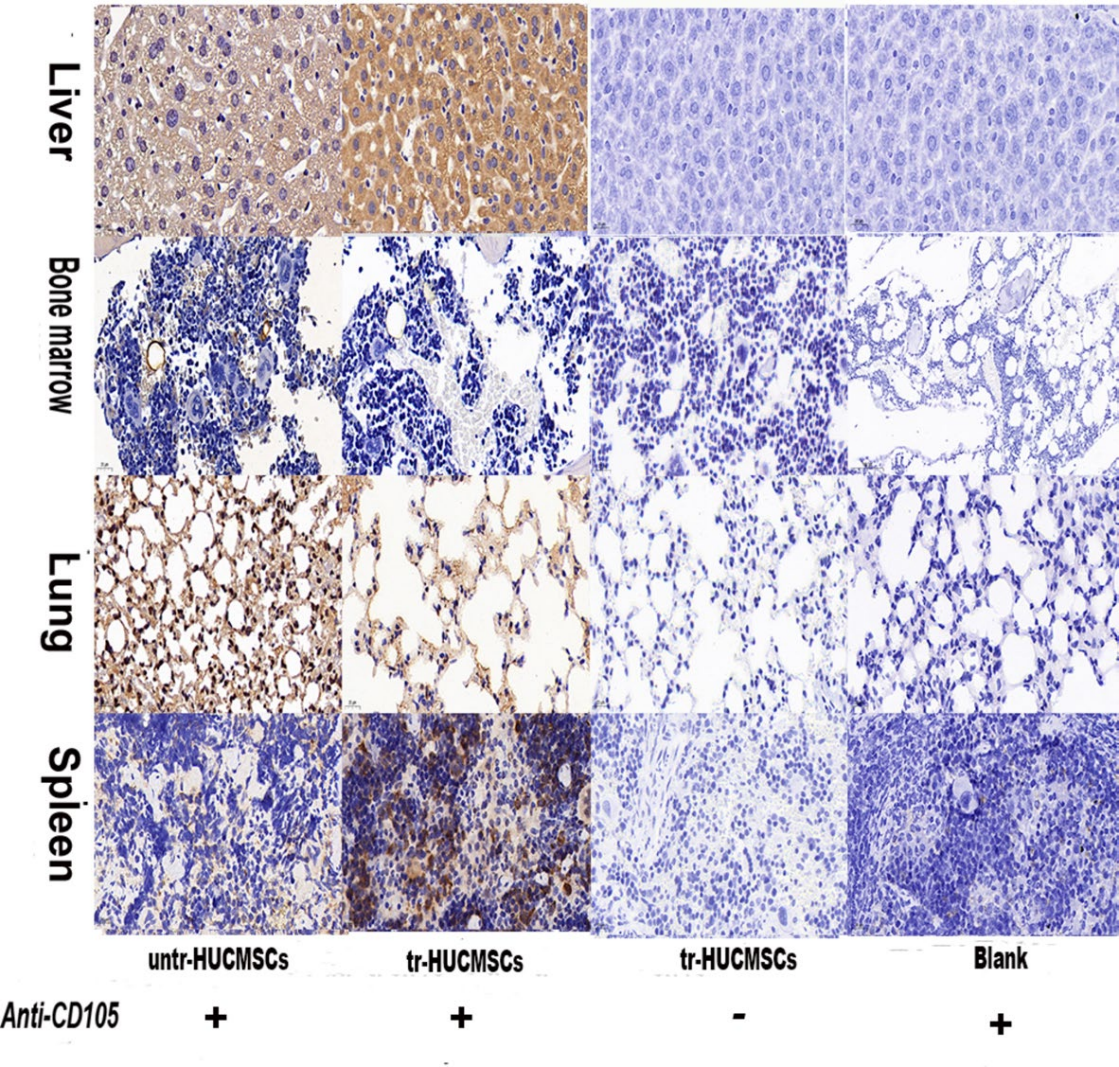
Immunohistochemical analysis of the distribution of tr-HUCMSCs or untr*-*HUCMSCs in NSG mice after 5 months transplantation. Selected animal tissues such as liver, spleen, lung and bone marrow were collected for immunohistochemical analysis (40×). (Blank: injected with 0.9% NS. Anti-CD105 +: adding anti-CD105 antibody; anti-CD105 -: no adding anti-CD105 antibody)

We also analyzed the distribution of tr-HUCMSCs or untr-HUCMSCs in NSG mice by the immunohistochemistry method. A mouse anti-human CD105 (a specific marker expressed on the membrane of HUCMSCs) antibody to identify human CD105 at both 2 weeks and 5 months post transplantation. At 2 weeks post transplantation, both tr-HUCMSCs and untr-HUCMSCs were observed in the spleen, liver, lung and bone marrow, with a particularly high concentration in the bone marrow compared to the control (Fig. 7). At 5 months post transplantation, weakly positive cells were detected in the spleen, liver, lung and bone marrow of both tr-HUCMSCs and untr-HUCMSCs groups compared to the control (Fig. 8).

## Discussion

AAV-mediated Gene therapy offers great potential for curing Hemophilia B. Targets the liver cells, which are primarily responsible for producing FIX, the traditional gene therapy has its advantages. however, it still raises concerns. For example, a substantial injection of the virus may lead to an inconsistent distribution of the virus across different cell types and may cause harm to other vital organs and systems, including the brain, the circulation system etc. Although side-effects by using AAV vectors have been generally acceptable in humans, additional research is still required to evaluate the safety of the virus as it transduce to different tissue cells including liver cells. The ultimate determinant of the success of traditional gene therapy may be the dose-dependent genotoxicity to other organs and tissues. The higher the vector dose, the higher the success rate of gene therapy; however, it also increases the likelihood of liver genotoxicity [16] and distribution to other tissues and system such as brain, circulation system etc. [27].

In this study, we developed a novel AAV vector expressing hFIXco, designated DJ/8-hFIX, which has potential applications in the treatment of hemophilia B. Our results demonstrate that tr-HL7702 exhibits robust hFIXco secretion in vitro, which was consistent with Hashimoto H reported that GFP-tagged DJ/8-hFIX effectively expresses GFP when compared to other wild-type serotypes [28].

Based on the previously reported normal AAV vector baseline used in gene therapy [29], we arbitrarily selected a dosage of 1 × 10^11^ vg/kg of pure LP1-hFIX for intravenous injection into mice tail veins as positive control. Fortunately, all *F9-KO* mice that received this dosage survived, and their coagulation activities were observed to be significantly improved. When compared to wild-type mice, minimal significance was observed between the *F9-KO* mice and wild-type mice. For future studies, we plan to increase the LP1-hFIX dosage for pure gene therapy and prepare for clinical trials.

MSCs are fibroblastic and plastic-adherent cells that possess the ability to differentiate into various mesenchymal lineages, both in vitro [30, 31] and in vivo [32, 33]. Sung Jin Kim et al. observed that scAAV2 and scAAV5 were effective in transferring the GFP gene to human bone marrow-derived MSCs and umbilical cord blood-derived MSCs [34]. AAV-DJ/8 demonstrates efficient transduction across various cell types derived from diverse species and tissues, including primary human hepatocytes, melanoma cells, and embryonic stem cells [28]. Yunxia Zhang et al. employed a lentiviral vector to transduce IL-21 into HUCMSCs, which were then transplanted into SKOV3 ovarian cancer xenograft-bearing nude mice. This approach offers a promising novel therapeutic strategy for the clinical management of ovarian cancer [35]. Given the immunogenic properties and self-renewal potential of HUCMSCs, we propose that HUCMSCs could serve as an excellent host cell for AAV mediated gene therapy to delivering hFIXco. To date, there are no known reports that have demonstrated the use of HUCMSCs as host cell in AAV mediated gene therapy in Hemophilia B applications.

In this study, We successfully transducted the DJ/8-hFIX into HUCMSCs as a substitute for high-dose vector injection, and the tr-HUCMSCs secreting comparable stability and continuity of hFIXco in vitro and in vivo. Although the results are still preliminary, it holds significant promise for potential clinical applications due to its preliminary yet promising nature. The ultimate utility of this approach necessitates further development through rigorous and extensive pre-clinical studies prior to clinical trial verification.

The AAV-mediated cell-based gene therapy exhibits a significant advantage over traditional gene therapy approaches. In animal studies, the total DJ/8-hFIX dose for cell-based gene therapy protocol was 1 × 10^9^ vg /kg. The total vector dosage used in this cell-based gene therapy was significantly lower than the traditional vector dosage (ranges from approximately 1 × 10^11^ to 1 × 10^12^ vg /kg). Lower dosages of vectors are employed in AAV-mediated cell-based gene therapy, simplifying the manufacturing processes and potentially leading to cost reduction in Hemophilia B treatment for future. Although this cell-based gene therapy has not yet been applied in humans, the significantly smaller total vector dosage used in this approach appears to offer greater safety compared to traditional gene therapy. By transducing the vectors into HUCMSCs in vitro without exposing them to other human tissue cells, our objective is to prevent inadvertent transduction of other tissue cells in vivo, thereby ensuring safety. HUCMSCs have been demonstrated to have minimal side effects in clinical trials. Our approach has the potential to avoid random AAV-mediated distribution to other tissues and system such as brain, circulation system etc. and also avoid liver genotoxicity induced by high-dose vector injection.

According to the report by Halder SK et al. [2], protein variants were most likely to be detrimental to protein structure and function, and further investigation is certainly needed into the structure of our purified hFIXco protein. However, studies in one Hemophilia B patient revealed that the activation of hFIXco secretion in tr-HUCMSCs is comparable with that of the positive control tr-HL7702. Although the observed increase in hFIX activity was relatively not very high which could be potentially attributed to the dilution of the cell supernatant, the elevation in FIX activity in this study is remarkable. Additionally, during this study, serum from a single Hemophilia B patient was obtained, and the results of coagulation time correction were provided an important evidence for its effectiveness. Despite the limited number of patients’ sera used, preliminary findings suggest that this study demonstrates, at least in part, that both tr-HUCMSCs and tr-HL7702 have the capacity to secrete hFIXco with coagulation correction potential.

Human MSCs were xenotransplanted into fetal sheep and integrated into different tissues including bone marrow, spleen, and liver [32]. Following a 2-week transplantation period, our experiments demonstrated the presence of HUCMSCs in bone marrow, spleen, and liver, particularly abundant in bone marrow, aligning with published reports [32]. This indicates that HUCMSCs maintain their functional properties, regardless of transduction status. Furthermore, tr-HUCMSCs may establish a foothold in the bone marrow microenvironment, exercising their secretory function for hFIXco. Following a five-month transplantation period, immunohistochemical analysis revealed the presence of weakly CD105-positive cells within the tissues. Hematoxylin-eosin staining confirmed similar tissue characteristics to the control group. This implies that the distribution of HUCMSCs or their transduced forms does not significantly impact tissue integrity, ruling out tumorigenic activity. The observed phenomenon may be attributed to the multilineage differentiation capabilities of HUCMSCs, facilitating their integration into the surrounding tissue.

Studies on cutting tail experiments of *F9-KO* mice revealed that the transgene hFIXco was effective in both direct injection and tr-HUCMSC treatment. The therapeutic effect of the transgene is achieved by minimizing bleeding episodes. Mazumder TH et al. [36] reported that evolutionary forces and genetic relationships can influence the codon utilization bias of a gene. The Calcium-binding EGF-like domain (residues 93–125), which may be concerned as crucial for FIX function, exhibits a high degree of similarity between hFIX and mFIX protein, differing by only 5 amino acids (supplementary Material 6). This may explain the therapeutic effect by shortened bleeding for *F9-KO* mice.

Giang N. Nguyen et al. [15] revealed AAV gene therapy in dogs with hemophilia A identifies several clonal expansions of transduced liver cells after 10 years observation. Our Hematoxylin-eosin staining results in NSG mice, however, did not show any clonal expansions of transduced cells in tissues including liver, spleen, lung, and brain after 5 months of experimentation. Although we did not investigate the genomic integrations in this study, the experiment may still provide some evidence for the safety of DJ/8-hFIX/ tr-HUCMSCs-mediated gene therapy.

A notable disparity was observed in the activity of hFIX detected in NSG mice that received injections of DJ/8-hFIX or 0.9% NS. This finding suggests that the detection of hFIX activity system, which was conducted in the human factor IX deficient plasma by employing a phospholipid (origin from rabbit brain powder) surface activator under the working concentration (0.025 mol/L) of CaCl_2_ is relatively specific to human FIX and is not much sensitive to mouse FIX. The results of human FIX activity in the sera of *F9-KO* mice 60 days post-injection were consistent with the findings obtained from NSG mice.

It is intriguing to observe that the *F9-KO* mice maintained excellent health throughout the experiment despite receiving tr-HUCMSCs without eliciting an immune response. The mechanism underlying the absence of an immune response against HUCMSCs in *F9-KO* mice remains enigmatic. One plausible explanation is the extremely low immunogenicity of HUCMSCs. Another potential factor could be the inherent characteristics of the *F9-KO* mice strain (C57BL/6JSmoc-F9^em1Smoc^). Report [37] cites the discovery of a mutation in the commercially available C57BL/6 strain, specifically gene duplication, that impairs the Dock2 gene and subsequently results in immune deficiency. The underlying mechanism of this phenomenon merits further investigation.

We have successfully transduced the HUCMSCs with the novel DJ/8-hFIX vector. Furthermore, a stable and consistent secretion of hFIXco was observed in the transduced HUCMSCs. The biological activity of secreted hFIXco was assessed both in vivo and in vitro. Notably, our study did not identify any significant differences between the direct injection of the DJ/8-hFIX vector and the one mediated via the HUCMSCs. Animal models have demonstrated the safety of this cell-based gene therapy, paving the way for its clinical application. While the disease-free survival observed at five months post-injection suggests the safety of HUCMSC-based DJ/8-hFIX gene therapy, and histological staining with hematoxylin-eosin reveals normal tissue morphology, the integration of the DJ/8-hFIX gene into host genomic DNA remains unclear. Additional research is required to investigate the presence of anti-DJ/8-hFIX antibodies in patients during clinical trials. The precise mechanism underlying the internalization of the DJ/8-hFIX vector by HUCMSCs and the secretion of active hFIXco remains enigmatic. Extensive research is necessary to elucidate this issue.

## Conclusion

We have discovered a novel and safer HUCMSCs mediated approach potentially effective for gene therapy in hemophilia B.

### Electronic supplementary material

Below is the link to the electronic supplementary material.

Supplementary Material 1

Supplementary Material 2

Supplementary Material 3

Supplementary Material 4

Supplementary Material 5

Supplementary Material 6

Supplementary Material 7

## Data Availability

The data used in this article are available from the corresponding author upon appropriate request.

## Abbreviations

MSCs: Mesenchymal stem cells
HUCMSCs: Human umbilical cord blood derived mesenchymal stem cells
FDA: The U.S. Food and Drug Administration
FIX: Coagulation factor IX
FVIII: Coagulation factor VIII
hFIXco: Codon-optimized human FIX
AAV: Adenovirus-associated virus
scAAV-DJ/8: self-complementary adeno-associated virus vector DJ/8
DJ/8-hFIX: scAAV-DJ/8-LP1-hFIXco vector
HEK293T: The human embryonic kidney 293T cell line
CHO: Chinese hamster ovary cell line
HL7702: Normal human liver cell line
FL: Human amnion cell line
ARDS: Acute respiratory distress syndrome
DMD: Duchenne’s muscular dystrophy
GVHD: Graft versus host disease
HAPO-HCR: The human apolipo-protein hepatic control region
hAAT: The human alpha-1-antitrypsin
HAPO-HCR-hAAT: Consecutive segments of HAPO HCR and hAAT
SV40: Modified SV40 small t antigen intron
Vg: Vector genomes
Vg/Kg: Vector genomes per Kilogram
s: Second
Kg: Kilogram
APTT: Activated Partial Thromboplastin Time
HBD: Heparin binding domain
tr-HUCMSCs: The DJ/8-hFIX transduced HUCMSCs
untr-HUCMSCs: The DJ/8-hFIX untransduced HUCMSCs
0.9% NS: 0.9% Normal Saline
tr-HL7702: The DJ/8-hFIX transduced HL-7702 cells
F9-KO: F9-Knock out mice
tr-CHO: The DJ/8-hFIX transduced CHO
tr-FL: The DJ/8-hFIX transduced FL cell lines
HRP: Horseradish peroxidase
NSG: NOD-SCID gamma mice
HBD: Heparin binding domain
DLEU2: Deleted in leukemia 2
IACUC: The Institutional Animal Care and Use Committee
ZJCLA: Zhejiang Center of Laboratory Animals

## References

[1] FDA Approves First Gene. Therapy to Treat Adults with Hemophilia B, Retrieved 11 2022, from https://www.businesswire.com/news/home/20221116005426/en/.

[2] Halder SK, Rafi MO, Shahriar EB, Albogami S, El-Shehawi AM, Daullah SMMU (2022). Identification of the most damaging nsSNPs in the human CFL1 gene and their functional and structural impacts on cofilin-1 protein. Gene.

[3] Bolous NS, Chen Y, Wang H, Davidoff AM, Devidas M, Jacobs TW (2021). Bolous the cost-effectiveness of gene therapy for severe hemophilia B: a microsimulation study from the United States perspective. Blood.

[4] Grimm D, Lee JS, Wang L, Desai T, Akache B, Storm TA (2008). In Vitro and in vivo gene therapy Vector Evolution via multispecies interbreeding and retargeting of Adeno-Associated viruses. J Virol.

[5] Gao GP, Alvira MR, Wang L, Calcedo R, Johnston J, Wilson JM (2002). Novel adeno-associated viruses from rhesus monkeys as vectors for human gene therapy. Proc Natl Acad Sci USA.

[6] Calcedo R, Vandenberghe LH, Gao G, Lin J, Wilson JM (2009). Worldwide epidemiology of neutralizing antibodies to adeno-associated viruses. J Infect Dis.

[7] Nathwani AC, Gray JT, Ng CY, Zhou J, Spence Y, Waddington SN (2006). Self complementary adeno-associated virus vectors containing a novel liver-specific human factor IX expression cassette enable highly efficient transduction of murine and nonhuman primate liver. Blood.

[8] Nathwani AC, Gray JT, McIntosh J, Ng CY, Zhou J, Spence Y (2007). Safe and efficient transduction of the liver after peripheral vein infusion of selfcomplementary AAV vector results in stable therapeutic expression of human FIX in nonhuman primates. Blood.

[9] Choi VW, Samulski RJ, McCarty DM (2005). Effects of adeno-associated virus DNA hairpin structure on recombination. J Virol.

[10] Haberman RP, Samulski RJ, McCown TJ (2003). Attenuation of seizures and neuronal death by adeno-associated virus vector galanin expression and secretion. Nat Med.

[11] Merten OW, Geny-Fiamma C, Douar AM (2005). Current issues in adeno-associated viral vector production. Gene Ther.

[12] Monahan PE, Samulski RJ (2000). AAV vectors: is clinical success on the horizon?. Gene Ther.

[13] Lek A, Wong B, Keeler A, Blackwood M, Ma K, Huang S (2023). Death after high-dose rAAV9 gene therapy in a patient with Duchenne’s muscular dystrophy. N Engl J Med.

[14] Chen X, Lim DA, Lawlor MW, Dimmock D, Vite CH, Lester T (2023). Biodistribution of Adeno-Associated Virus Gene Therapy following Cerebrospinal Fluid-Directed Administration. Hum Gene Ther.

[15] Nguyen GN, Everett JK, Kafle S, Roche AM, Raymond HE, Leiby J (2021). A long-term study of AAV gene therapy in dogs with hemophilia A identifies clonal expansions of transduced liver cells. Nat Biotechnol.

[16] Bell P, Wang L, Lebherz C, Flieder DB, Bove MS, Wu D (2005). No evidence for tumorigenesis of AAV vectors in a large-scale study in mice. Mol Ther.

[17] Li H, Malani N, Hamilton SR, Schlachterman A, Bussadori G, Edmonson SE et al. Assessing the potential for AAV vector genotoxicity in a murine model.Blood. 2011;117(12):3311–9. 10.1182/blood-2010-08-302729 PMC3069673 21106988

[18] Nault JC, Datta S, Imbeaud S, Franconi A, Mallet M, Couchy G (2015). Recurrent AAV2-related insertional mutagenesis in human hepato cellular carcinomas. Nat Genet.

[19] Manochantr S, U-pratya Y, Kheolamai P, Rojphisan S, Chayosumrit M, Tantrawatpan C (2013). Immunosuppressive properties of mesenchymal stromal cells derived from amnion, placenta, Wharton’s jelly and umbilical cord. Intern Med J.

[20] El Omar R, Beroud J, Stoltz JF, Menu P, Velot E, Decot V (2014). Umbilical cord mesenchymal stem cells: the new gold standard for mesenchymal stem cell-based therapies?. Tissue Eng Part B Rev.

[21] Fan CG, Zhang QJ, Zhou JR. Therapeutic potentials of mesenchymal stem cells derived from human umbilical cord. Stem Cell Rev 2011:7195–207. 10.1007/s12015-010-9168-8 20676943

[22] Zhao L, Chen S, Yang P, Cao H, Li L (2019). The role of mesenchymal stem cells in hematopoietic stem cell transplantation: prevention and treatment of graft-versus-host disease. Stem Cell Res Ther.

[23] Nathwani AC, Davidoff A, Hanawa H, Zhou JF, Vanin EF, Nienhuis AW (2001). Factors influencing in vivo transduction by recombinant adeno-associated viral vectors expressing the human factor IX cDNA. Blood.

[24] Nathwani AC, Rosales C, McIntosh J, Rastegarlari G, Nathwani D, Raj D (2011). Long-term safety and efficacy following systemic administration of a self-complementary AAV vector encoding human FIX pseudotyped with serotype 5 and 8 capsid proteins. Mol Ther.

[25] Haas J, Park EC, Seed B (1996). Codon usage limitation in the expression of HIV-1 envelope glycoprotein. Curr Biol.

[26] Hildinger M, Baldi L, Stettler M, Wurm FM (2007). High-titer, serum-free production of adeno-associated virus vectors by polyethyleneimine-mediated plasmid transfection in mammalian suspension cells. Biotechnol Lett.

[27] Sun K, Liao MZ (2022). Clinical pharmacology considerations on recombinant Adeno-Associated Virus-based gene therapy. J Clin Pharmacol.

[28] Hashimoto H, Mizushima T, Chijiwa T, Nakamura M, Suemizu H (2017). Efficient production of recombinant adeno-associated viral vector, serotype DJ/8, carrying the GFP gene. Virus Res.

[29] George LA, Sullivan SK, Giermasz A, Rasko JEJ, Samelson-Jones BJ, Ducore J (2017). Hemophilia B Gene Therapy with a high-specific-activity factor IX variant. N Engl J Med.

[30] Horwitz EM, Le Blanc K, Dominici M, Mueller I, Slaper-Cortenbach I, Marini FC (2005). Clarification of the nomenclature for MSC: the International Society for Cellular Therapy position statement. Cytotherapy.

[31] Ding DC, Shyu WC, Lin SZ (2011). Mesenchymal stem cells. Cell Transpl.

[32] Liechty KW, MacKenzie TC, Shaaban AF, Radu A, Moseley AM, Deans R (2000). Human mesenchymal stem cells engraft and demonstrate site-specific differentiation after in utero transplantation in sheep. Nat Med.

[33] Anjos-Afonso F, Siapati EK, Bonnet D (2004). In vivo contribution of murine mesenchymal stem cells into multiple cell-types under minimal damage conditions. J Cell Sci.

[34] Kim SJ, Lee WI, Heo H, Shin O, Kwon YK, Lee H (2007). Stable gene expression by self-complementary adeno-associated viruses in human MSCs. Biochem Biophys Res Commun.

[35] Zhang Y, Wang J, Ren M, Li M, Chen D, Chen J (2014). Gene therapy of ovarian cancer using IL-21-secreting human umbilical cord mesenchymal stem cells in nude mice. J Ovarian Res.

[36] Mazumder TH, Alqahtani AM, Alqahtani T, Emran TB, Aldahish A, Uddin A (2021). Analysis of Codon usage of Speech Gene FoxP2 among Animals.Biology. (Basel).

[37] Mahajan VS, Demissie E, Mattoo H, Viswanadham V, Varki A, Morris R (2016). Striking immune phenotypes in diverse gene-targeted mice are driven by a copy number variant originating from a commercially available C57BL/6 strain. Cell Rep.

