# Supplementary material for: Human umbilical cord mesenchymal stem cell-based gene therapy for hemophilia B using scAAV-DJ/8-LP1-hFIXco transduction
Source: Stem Cell Res Ther. 2024 Jul 18;15:210. doi: 10.1186/s13287-024-03824-y (PMC11256413; doi:10.1186/s13287-024-03824-y)
Supplement: Supplementary file 1 — Supplementary Material 1 [file 13287_2024_3824_MOESM1_ESM.pdf]

浙江省实验动物中心实验动物福利伦理审查同意书

Approval of Animal Use Protocol, IACUC, ZJCLA

**批准编号**<br>**Approval No.**	ZJCLA-IACUC-20020133
实验名称（中英文）<br>Protocol Title	ScAAV-DJ/8-LP1-hFIXco载体转导的间充质干细胞在血友病乙小鼠基因治疗的研究<br>（Mesenchymal Stem Cell-Based Gene Therapy for Hemophilia B using scAAV-DJ/8-LP1-hFIXco Transduction）
**申请人**<br>**Applicant**	卜梓斌<br>Zibin.Bu
**申请单位**<br>**Application Institution**	浙江大学医学院附属儿童医院<br>Division/Center of Hematology-oncology, Children’s Hospital, Zhejiang University School of Medicine
**申请材料**<br>**Applicant Documents**	动物实验伦理审查申请材料是否齐全？是 【√】，否【 】<br>Are the application documents complete?　YES【√】 NO【 】
**审查意见**<br>**Results of Review**	【√】符合动物福利伦理要求，同意实验 Agree<br>【 】调整方案后，可进行实验 Agree after modification

本动物实验方案经过实验动物福利伦理委员会审核，符合动物保护、动物福利和伦理原则，符合国家实验动物福利伦理的相关规定。

The animal use protocol listed below has been reviewed and approved by the Institutional Animal Care and Use Committee (IACUC), ZJCLA.

浙江省实验动物中心实验动物福利伦理委员会（IACUC, ZJCLA）

主席（Chairman）：[illegible]

日期（Date）： 2022.9.15

地址：浙江省实验动物中心，杭州市天目山路182号(暨杭州医学院实验动物中心)，　邮编：310013 邮箱：13067719943@163.com
Add: Zhejiang Center of Laboratory Animals , No.182 Tianmushan Road (Hangzhou Medical College Laboratory Animal Center) , Hangzhou, 310013, P.R. China, E-MAIL: 13067719943@163.com
