# Supplementary material for: Human umbilical cord mesenchymal stem cell-based gene therapy for hemophilia B using scAAV-DJ/8-LP1-hFIXco transduction
Source: Stem Cell Res Ther. 2024 Jul 18;15:210. doi: 10.1186/s13287-024-03824-y (PMC11256413; doi:10.1186/s13287-024-03824-y)
Supplement: Supplementary file 3 — Supplementary Material 3 [file 13287_2024_3824_MOESM3_ESM.pdf]

临床科研一般试验医学伦理审查批件

Approval Letter of Ethics Committee

（伦）审编号：2015-IRB-023

<table>
<tr><td>项目名称</td><td colspan="6">儿童出血性疾病及血液肿瘤用残余标本的深低温保存及回顾性研究</td></tr>
<tr><td>研究类型</td><td colspan="2">回顾性研究</td><td colspan="2">申请专业</td><td colspan="2">血液肿瘤</td></tr>
<tr><td>审查批准文件</td><td colspan="6">1. 临床科研一般试验研究者声明书
2. 主要研究者资质（研究者简历）
3. 试验研究方案 版本日期：版本日期：20150225 版本号：第 1.0 版
4. 知情同意书（ICF）版本日期：版本日期：20150226 版本号：第 1.1 版
5. 研究小组名单及分工</td></tr>
<tr><td>申办单位/科室</td><td colspan="2">浙江大学医学院附属儿童医院血液科</td><td colspan="2">项目负责人</td><td colspan="2">汤永民</td></tr>
<tr><td>出席人数</td><td colspan="2">应到：23 人</td><td colspan="2">实到：16 人</td><td colspan="2">回避：0 人</td></tr>
<tr><td rowspan="2">投票结果</td><td>同意</td><td>作必要修正后同意</td><td>作必要修正后重审</td><td>不同意</td><td colspan="2">终止或暂停已批准的试验</td></tr>
<tr><td>12</td><td>4</td><td>0</td><td>0</td><td colspan="2">0</td></tr>
</table>

审查方式：会议审查，快速审查

评审决定：

根据中华人民共和国国家食品药品监督管理总局 2003 年颁布实施的《药物临床试验质量管理规范》以及《赫尔辛基宣言》等相关伦理指导原则，本伦理委员会于 **2015 年 2 月 26 日 下午** 在行政楼 **3 楼会议室** 经**会议审查** 决定**同意按试验研究方案进行研究，知情同意书作必要修正后同意**。会后，项目申请人修改的知情同意书，经主审快速审查后符合伦理委员会的要求，同意项目按修正后的知情同意书进行研究。

伦理跟踪审查频率：12 个月

伦理委员会主任或副主任签名： 日期 2015-2-27

（浙江大学医学院附属儿童医院医学伦理委员会）盖章

附件：1.伦理委员名单及签到表

伦理委员会名单

姓名	任职	性别	专业	工作单位	职称/职务	签到/备注
俞惠民	主任委员	男	儿科学/新生儿	浙江大学医学院儿童医院	教授/主任医师	
陈志敏	副主任委员	男	儿科学/小儿呼吸	浙江大学医学院儿童医院	教授/主任医师	
赵正言	副主任委员	男	儿科学/儿童保健	浙江大学医学院儿童医院	教授/主任医师	/
杜立中	委员	男	儿科学/新生儿	浙江大学医学院儿童医院	教授/主任医师	
舒　强	委员	男	儿外科/胸心外科	浙江大学医学院儿童医院	教授/主任医师	
章伟芳	委员	女	行政管理	浙江大学医学院儿童医院	副研究员	
龚方戚	委员	男	儿科学/心脏病	浙江大学医学院儿童医院	教授/主任医师	
江克文	委员	男	儿科学/神经内科	浙江大学医学院儿童医院	主任医师	
傅君芬	委员	女	儿内科/内分泌	浙江大学医学院儿童医院	教授/主任医师	
徐美春	委员	女	儿科学/急诊医学	浙江大学医学院儿童医院	主任医师	
江米足	委员	男	儿内科/消化	浙江大学医学院儿童医院	教授/主任医师	
梁建凤	委员	男	卫生统计学	浙江大学医学院儿童医院	统计师	
王　珏	委员	女	药学	浙江大学医学院儿童医院	主任药师	
尚世强	委员	男	实验检验	浙江大学医学院儿童医院	主任医师	
楼晓芳	委员	女	儿科护理	浙江大学医学院儿童医院	主任护师	
陈洁	委员	女	儿内科/消化	浙江大学医学院儿童医院	主任医师	
施丽萍	委员	女	小儿重症医学	浙江大学医学院儿童医院	主任医师	
倪韶青	委员	女	药学	浙江大学医学院儿童医院	副主任药师	
谈林华	委员	女	小儿重症医学	浙江大学医学院儿童医院	主任医师	
崔满兴	委员	男	法律	上海锦天城（杭州）律师事务所	律师	
韩根跃	委员	男	社区工作	杭州下城区凤麟社区	社区主任	
施卫星	委员	男	卫生伦理	浙江大学医学院公卫学院	教授	
沈立宏	委员	女	学前教育	杭州市下城区麒麟幼儿园	幼教	

秘书：漆林艳、丁雯

浙江大学医学院附属儿童医院医学伦理委员会　　盖章　　日期：2015.02.26

（联系地址：浙江省杭州市竹竿巷57号　　电话：0571-87060205）
