# Supplementary figures and images for: Human umbilical cord mesenchymal stem cell-based gene therapy for hemophilia B using scAAV-DJ/8-LP1-hFIXco transduction
Source: Stem Cell Res Ther. 2024 Jul 18;15:210. doi: 10.1186/s13287-024-03824-y (PMC11256413; doi:10.1186/s13287-024-03824-y)

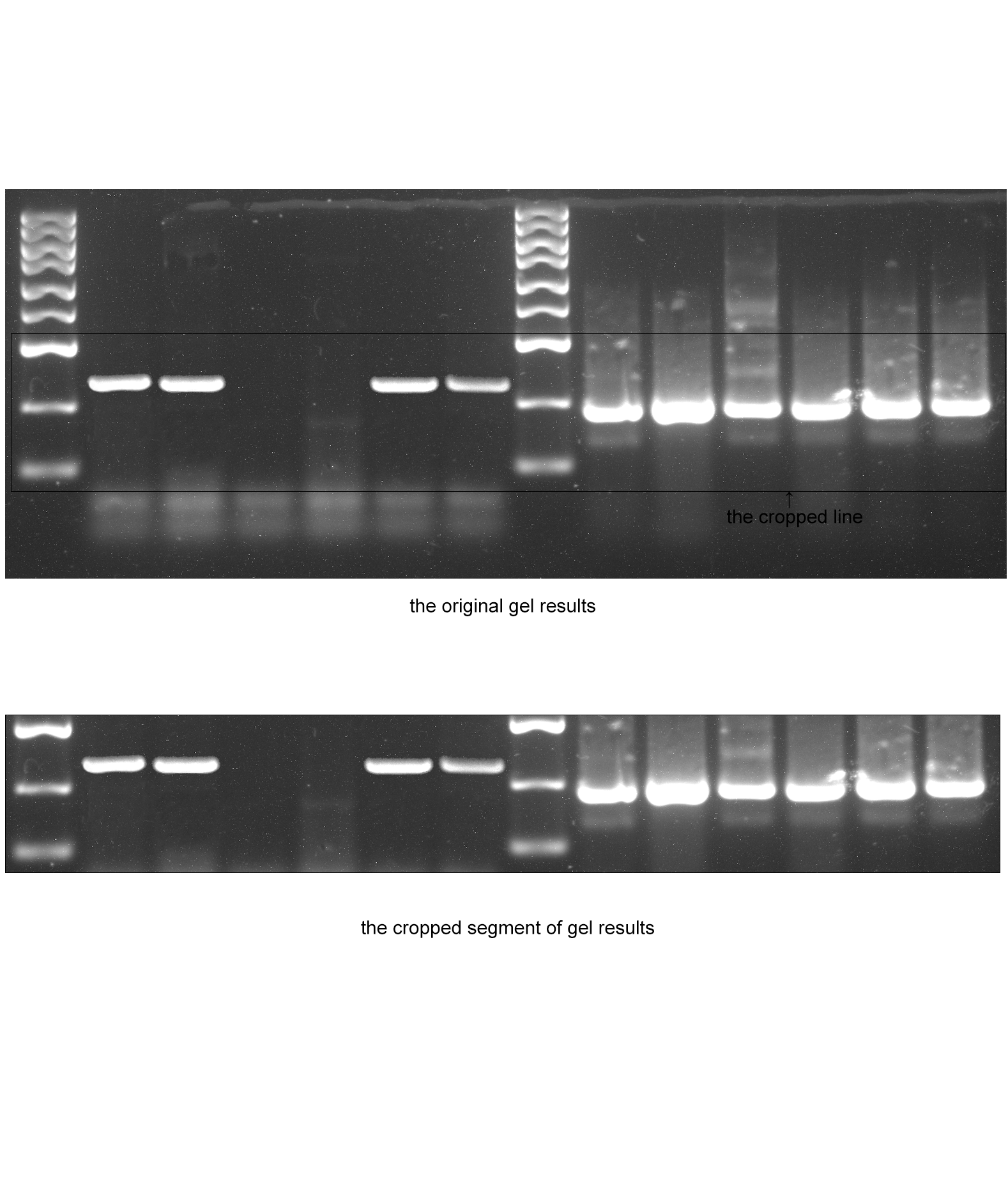

Supplement: Supplementary file 4 — Supplementary Material 4 [file 13287_2024_3824_MOESM4_ESM.tif]

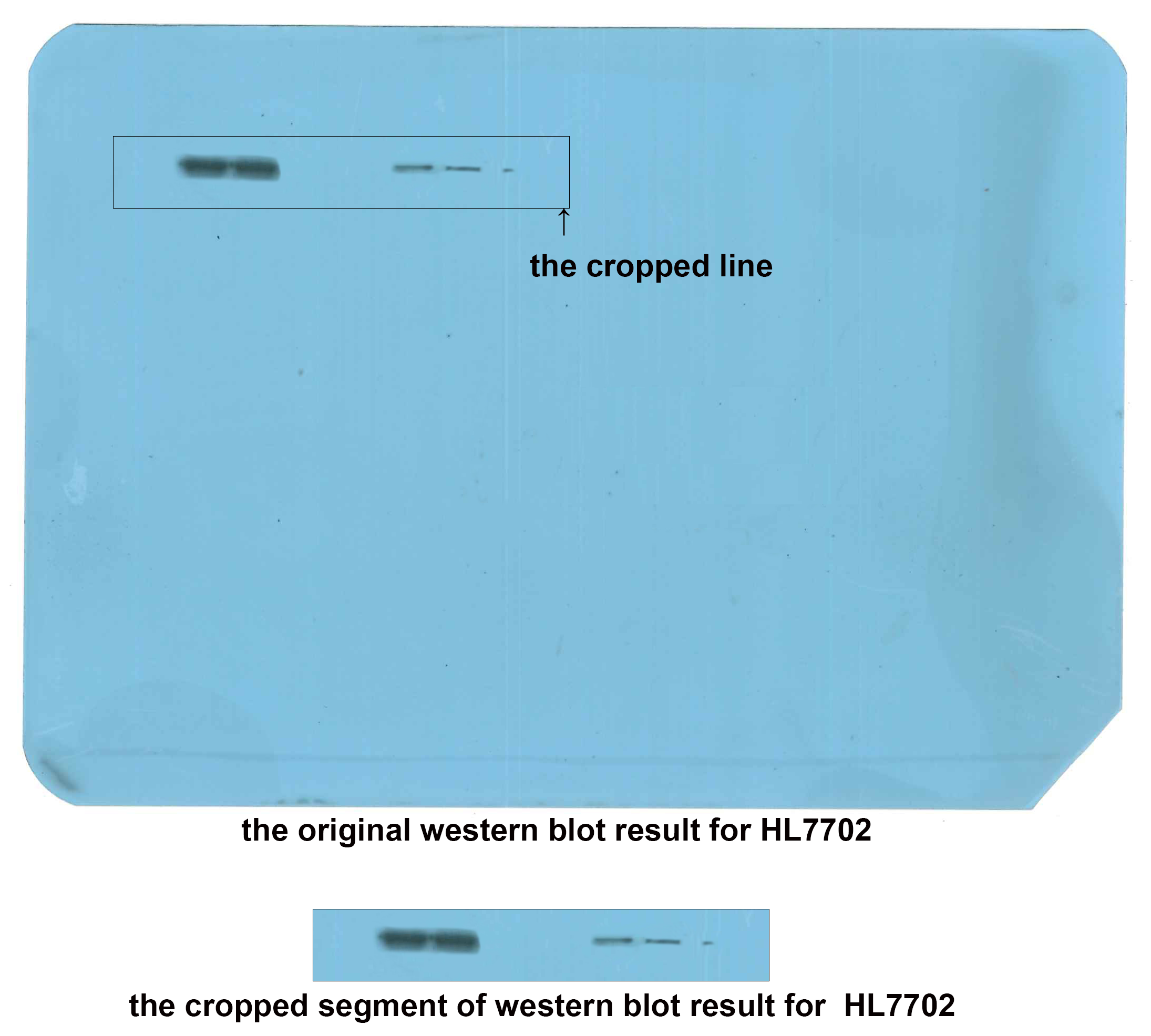

Supplement: Supplementary file 5 — Supplementary Material 5 [file 13287_2024_3824_MOESM5_ESM.tif]

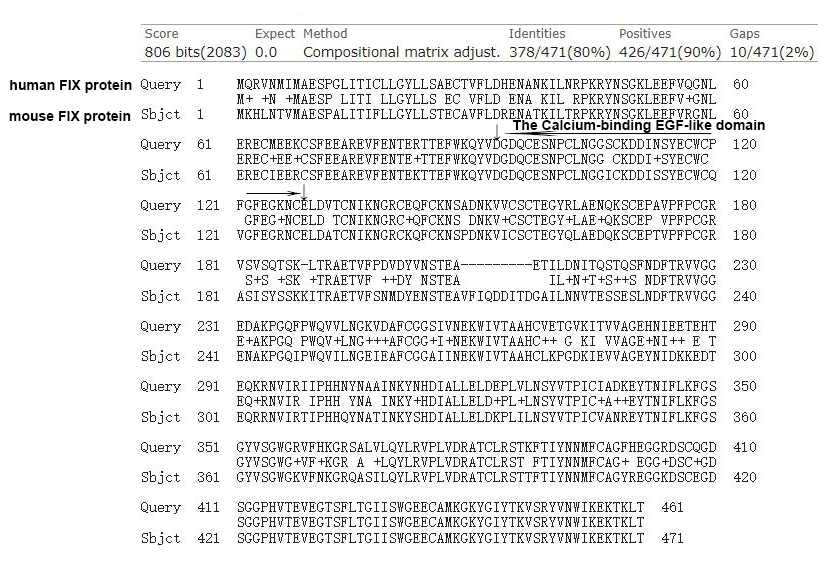

Supplement: Supplementary file 6 — Supplementary Material 6 [file 13287_2024_3824_MOESM6_ESM.tif]

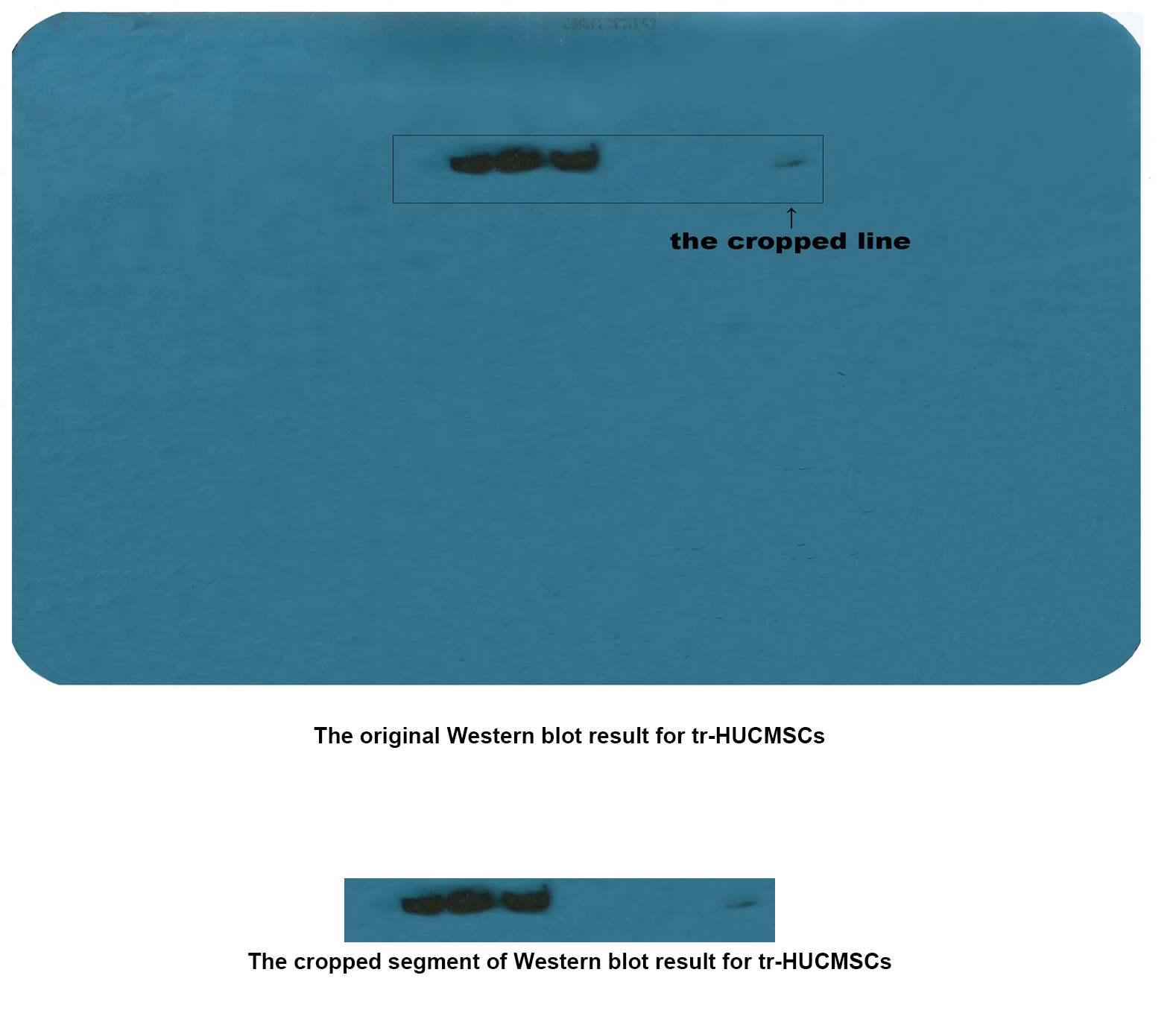

Supplement: Supplementary file 7 — Supplementary Material 7 [file 13287_2024_3824_MOESM7_ESM.tif]
