# Supplementary material for: Human umbilical cord mesenchymal stem cell-based gene therapy for hemophilia B using scAAV-DJ/8-LP1-hFIXco transduction
Source: Stem Cell Res Ther. 2024 Jul 18;15:210. doi: 10.1186/s13287-024-03824-y (PMC11256413; doi:10.1186/s13287-024-03824-y)
Supplement: Supplementary file 2 — Supplementary Material 2 [file 13287_2024_3824_MOESM2_ESM.doc]

Answering letter

Dear Editor:

Thank you and our reviewers very much for your careful considering our manuscript. We have carefully read the questions and comments raised by our reviewers and try to answer the questions point by point. We carefully revised the manuscript and highlight all changes made when revising the manuscript. The followings are the questions raised by our reviewers and our answers to each question.

Reviewer #1: For authors
The authors developed a cell-based gene therapy utilizing the scAAV-DJ/8-LP1-hFIXco vector to transduce human umbilical cord blood-derived mesenchymal stem cells (HUCMSCs) for treating hemophilia B. This innovative therapy holds promise for reducing the side effects and costs associated with the conventional direct injection of AAV5 virus particles containing the gene encoding coagulation factor IX. However, before publication, several issues need to be addressed.
Answer: Thank you very much for your nice comments about our work.
Major:
1. Does the DJ/8-hFIX gene integrate into the genomic DNA of HUCMSCs, or does it merely exist in the HUCMSCs as episomes? If it integrates into the genomic DNA, does it integrate into safe sites such as the AAVS1 locus (chromosome 19 q13.42)? If it exists in the cell as episomes, why does hFIX persist for five months? If it exists in the cell as episomes, why are the authors so worried about the tumorigenicity?

Answer: Thank you for your questions. We are not very worried about the tumorigenicity, however the safety of biotherapy/gene therapy to recipients are always the concerns of research. Gene therapy has experienced a very long time to find out the fact that the AAV vector is suitable for gene delivery. So AAV based gene therapy has been generally accepted as the safer approach. Our scAAV-DJ/8-LP1-hFIXco vector should be as the same as what have been reported in the literature. It is reasonable to expect that our vector should exist in the HUCMSCs as episomes in most situations, although it may also integrate into genomic DNA in rare cases because the FIX activity was quite stable and non-tumorigenicity was found even after 5 months observation in mice. So although we temperately did not investigate the details of genomic integrations in this study, we plan to accomplish this experiment to address this question in our future study. In this manuscript, we have added the paragraph in the introduction part as follows: Giang N. Nguyen et al^14^ revealed AAV gene therapy in dogs with hemophilia A identifies clonal expansions of transduced liver cells. They identified a residential AAV vector including a whole F VIII gene integrated in gene DLEU2 (deleted in leukemia 2) in one dog. The integration of the host genome has been extensively studied in hemophilia B gene therapy research^15-17^, demonstrating its safety through numerous experiments. Random integration into the host genome may indeed occur. The concern regarding tumorigenicity associated with gene therapy remains a persistent issue of concern.

We also have added the paragraph in discussion as follows: Giang N. Nguyen et al^14^ revealed AAV gene therapy in dogs with hemophilia A identifies several clonal expansions of transduced liver cells after 10 years observation. Our Hematoxylin-eosin staining results in NSG mice, however, did not show any clonal expansions of transduced cells in tissues including liver, spleen, lung, and brain after 5 months of experimentation. Although we did not investigate the genomic integrations in this study, the experiment may still provide some evidence for the safety of DJ/8-hFIX/ tr-HUCMSCs-mediated gene therapy.

2. For the F9-KO study (page 14, lines 9-23), there is no figure associated with this study.

Answer: Thank you for this suggestion. We added the information and rearranged the data in the Fig 2. (please see the detail in the Fig 2)
3. Why is there no immune response against HUCMSCs in F9-KO mice? Were the mice treated with immune-suppressor drugs?

Answer: Thank you for the question. We did not use the immune-suppressor drugs for the experiment. In the whole experiment the F9-KO mice seemed very healthy after being treated with HUCMSCs. There was no serious or only minor immunoreaction happened during the experiment. We don’t know why there is no immune response against HUCMSCs in F9-KO mice. The reasonable explanation for this phenomenon may attribute to the very low immunogenicity of the HUCMSCs to mouse. Another reason may exist is the *F9*-Knock out (F9-KO) mice ( strain name: C57BL/6JSmoc-F9^em1Smoc^) itself. According to Mahajan VS reported in the article “Striking immune phenotypes in diverse gene-targeted mice are driven by a copy number variant originating from a commercially available C57BL/6 strain” (Cell Rep. 2016; 31;15(9):1901-9）, the author discovered a mutation called gene duplication in a commercially available C57BL/6 strain, which disrupts the function of the *Dock2* gene, leading to immune deficiency. The underlying mechanism of this phenomenon merits further investigation.
4. Throughout the document, the manuscript does not present the advantages of tr-HUCMSCs over DJ/8-hFIX in the NGS and F9-KO mice.

Answer: Thanks for this question. The advantages of tr-HUCMSCs over DJ/8-hFIX we mentioned in discussion part, but not very clearly described in more detail. Now we have added the contents in the discussion part as follows: The AAV-mediated cell-based gene therapy exhibits a significant advantage over traditional gene therapy approaches. In animal studies, the total DJ/8-hFIX dose for cell-based gene therapy protocol was 1×10^9^ vg /kg. The total vector dosage used in this cell-based gene therapy was significantly lower than the traditional vector dosage (ranges from approximately 1×10^11^ to 1×10^12^ vg /kg). Lower dosages of vectors are employed in AAV-mediated cell-based gene therapy, simplifying manufacturing processes and potentially leading to cost reduction in Hemophilia B treatment for future. Although this cell-based gene therapy has not yet been applied in humans, the significantly smaller total vector dosage used in this approach appears to offer greater safety compared to traditional gene therapy. By transducing the vectors into HUCMSCs in vitro without exposing them to other human tissue cells, our objective is to prevent inadvertent transduction of other tissue cells in vivo, thereby ensuring safety. HUCMSCs have been demonstrated to have minimal side effects in clinical trials. Our approach has the potential to avoid potential liver, brain toxicity that can be induced by high-dose vector injection.

5. On page 17, line 46, the authors mention, "To date, there are no known reports that have demonstrated the use of HUCMSCS as host cell in gene therapy applications.". Please ensure this statement is accurate by checking additional references and discussing other reports in the Discussion section.

Answer: Thank you for your important question and advice. Indeed, the utilization of HUCMSCS as a host cell in gene therapy applications by using lentiviral vector for ovarian cancer has been previously demonstrated. However, there is currently a lack of published reports demonstrating the use of HUCMSCS as a host cell in AAV mediated gene therapy applications for Hemophilia B. We added it in the discussion part.

Minor
1. In the Methods section, please provide more detail about the activity assay of hFIXco. Additionally, could you clarify the definition of "100%" in this context?

Answer: Thank you for these questions, the hFIX activity assay we described in our original manuscript was too simple, now we added the detailed protocol to the method section as follows: The APTT assay, a one-stage clotting factor assay, was conducted in the human factor IX deficient plasma by employing a phospholipid surface activator (made from rabbit brain powder) under the working concentration of CaCl_2_ (0.025mol/L). The human factor IX deficient plasma was provided by Siemens (Germany). The analysis was performed using a fully automated coagulation analyzer (CS-5100 system, Sysmex Corporation, Japan). FIX:C was estimated using a reference curve prepared from standard human plasma (Siemens Healthcare GmbH, Germany), which was calibrated by the manufacturer against the WHO international standard for FIX. The calibrator signed the FIX percent was also provided by Siemens (Germany).
2. What is the similarity between hFIX and mFIX? Why is hFIX activity specific to human FIX and does not cross-react with mouse FIX (page 20, line 12)? Furthermore, how can hFIX exert a therapeutic effect, as observed by the shortened bleeding time in F9-KO mice?

Answer: Thank you for the critical questions. The similarity between hFIX and mFIX protein is as follows:

From the comparison result between hFIX and mFIX, the two proteins shared 97.9% of similarity. And the similarity of Calcium-binding EGF-like domain which may be concerned as crucial for FIX function, was from protein sequence within 93-125 with only 5 amino acid difference，which may explain the therapeutic effect by shortened bleeding for F9-KO mice. Interestingly enough, when we determined the hFIXco activity in the mouse serum, the hFIXco activity was clearly measured without cross reaction to mFIX identified by using the human FIX activity assay. That may be explained by the whole human FIX detection system designed by Siemens company are relatively specific for human FIX while it is not much sensitive to mouse FIX. We are sorry for not clearly describing this. In our revised manuscript, we have amended the description as follows: This finding suggests that the detection of hFIX activity system, which was conducted in the human factor IX deficient plasma by employing a phospholipid (origin from rabbit brain powder) surface activator under the working concentration (0.025mol/L) of CaCl_2_ is relatively specific to human FIX and it is not much sensitive to mouse FIX.

3.Please provide the full name of activated partial thromboplastin time (APTT) the first time it appears. Additionally, could you add the APTT protocol to the Methods section?

Answer: Thank you very much for your suggestion. We have added the APTT protocol to the Methods section and provided the full name of activated partial thromboplastin time (APTT) the first time it appears. protocol：The APTT assay, a one-stage clotting factor assay, was conducted in the human factor IX deficient plasma by employing a phospholipid surface activator (made from rabbit brain powder) under the working concentration of CaCl2 (0.025mol/L).

4. Please provide the Institutional Review Board (IRB) information because Fig-2b involved one hemophilia B patient serum.

Answer: The hemophilia B patient serum utilized in our study originated from residual samples collected during routine coagulation function tests conducted during clinical visits. These samples were part of a cryopreservation and retrospective study aimed at diagnosing hematology and oncology conditions among children. The use of these samples was approved by our institutional ethnic review board under approval number 2015-IRB-Q.5.

Please provide n value for Fig-2a.

Answer: Thank you for your advice. We have added the n value for Fig-2a.

6. In Fig-4a, why does the activity of FIX fluctuate several times during the five months of culture?

Answer: Thanks for your question. In five month’s culture, the reason why the activity of FIX fluctuate several times may attribute to the cell culture. Despite the cells seeded at an appropriate number, we could not exclude the possibility that the fluctuation of the FIX activity was possibly caused by normal variation of our FIX activity measuring assay.

In Fig-2, the text sizes of a and b are different. Additionally, please indicate the unit of the Y-axis for the APTT assay.

Answer: Thanks for your careful review and pointing out this. We have rearranged the Figure, and uniform the font (please see the revised Fig 2). The unit of the Y-axis for APTT assay is second (s) which has been added in our revised manuscript
8. In Fig-3b, why is the protein expression level of hFIX in tr-HL7702 cells not shown, particularly in the supernatant of cell lysate after 24 hours or five months of culture?

Answer: Thank you for your question. We did the experiment, and now we have added the results to the Figure 3 in our revised manuscript. The results are as follows：

Fig 3. **The expression of hFIXco in tr-HUCMSCs and tr-HL7702 by PCR and Western blot.** (a) hFIXco RNA was detected in tr-HUCMSCs and HL7702 using RNA detection techniques (the figure presented is the cropped gel. Full-length gels are presented in Supplementary Figure 1). The gel lanes were labeled as follows: Lane 1: marker, Lane 2 and Lane 3: tr-HUCMSCs cultured for 24 hours and 5 months respectively, Lane 4 and Lane 5: untr-HUCMSCs cultured for 24 hours and 5 months, respectively, Lane 6 and Lane 7: tr-HL7702 cultured for 24 hours and 5 months, respectively. Lane 8: marker. Lanes 9-14: GAPDH served as an internal control in the above-mentioned samples. (b) the FIX protein detection in the supernatants of tr-HL7702 (the figure presented is the cropped blot. The full-length blot is presented in Supplementary Figure 2). The samples were loaded as follows: Lane 1: marker, Lane 2 and Lane 3: supernatants of tr-HL7702 after 24 hours and 5 months culture, Lane 4 and Lane 5: supernatants of tr-CHO after 24 hours and 5 months culture, Lane 6 and Lane 7: supernatants of HL7702 after 24 hours and 5 months culture, Lane 8: pure FIX protein (8ng/lane). (c) the hFIXco protein detection in the supernatants and cell lysates of tr-HUCMSCs (the figure presented is the cropped blot. The full-length blot is presented in Supplementary Figure 3). The samples were loaded as follows: Lane 1: marker, Lane 2 and Lane 3: supernatants of tr-HUCMSCs after 24 hours and 5 months culture, Lane 4: cell lysate of tr-HUCMSCs after 5 months of transduction, Lane 5 and Lane 6: supernatants and cell lysate of untr-HUCMSCs for 24 hours, Lane 7: blank medium. Lane 8: pure FIX protein (8ng/lane).

9. On page 30, line 53, "DJ/8-hIX" should be replaced with "DJ/8-hFIX"

.Answer: Thank you very much for your careful review, we have corrected this error.
10. On page 23, line 13, increase the text size for "HAPO-HCR-hAAT" to match the surrounding text.

Answer: Thank you very much for your suggestion. We have adjusted the text size.
11. On page 18, line 36, "this" should be capitalized: "This."

Answer: Thank you very much. We have corrected the error.
12. On page 22, line 9, the authors mention, "Further investigation is needed to determine the presence of anti-DJ/8-hFIX antibodies in patients during clinical trials.". Could you please provide more details about this concern? Why is further investigation necessary?
Answer: From clinical point of view, in some severe case of hemophilia B, repeated use of FIX will cause inhibitor (anti-FIX antibody) generation which may compromise the therapeutic effects of FIX replacement therapy. The therapy provided by our approach may also cause the same situation. The antibody monitoring can only be investigated during clinical trial.

Reviewer #2: 1. Are there controversies in this field? What are the most recent and important achievements in the field? In my opinion, answers to these questions should be emphasized. Perhaps, in some cases, novelty of the recent achievements should be highlighted by indicating the year of publication in the text of the manuscript.

Answer: Thank you very much for your questions and suggestions. Gene therapy has a lengthy history, during which the AAV vector has been identified as a relatively safe vector for gene delivery. Nonetheless, the field continues to harbor ongoing debates and controversies. Firstly, concerns persist regarding the safety of gene therapy. AAV vectors primarily reside in target cells as episomes, existing as either circular genomes or concatemers, and occasionally integrate into the host genome. Numerous experiments in hemophilia B gene therapy research have demonstrated the safety of host genome integration. Concerns over tumorigenicity associated with gene therapy continue to persist as a significant issue. Secondly, there is a risk that liver-targeted AAV-mediated gene therapy may unintentionally target non-target organs, including the brain. Thirdly, the extremely high cost of gene therapy may significantly limit its widespread application. In November 2022, the U.S. Food and Drug Administration (FDA) granted approval for the first gene therapy medication, Hemgenix, for treating adult patients with hemophilia B. This therapy utilizes AAV5 as the vector to deliver the FIX variant padua 1. CSL Behring, an Australian drug manufacturer, has priced a single dose of Hemgenix at $3.5 million, thereby establishing it as the most costly drug globally. We have revised the text in the introduction section.

2. The results and discussion section is very weak and no emphasis is given on the discussion of the results like why certain effects are coming in to existence and what could be the possible reason behind them?

Answer: Thanks for your questions and suggestions. we have rewritten many sentences in the results and discussion parts. In results part, we highlighted the presence of healthy life during the whole experiment and non-clonal expansions of transduced cells in tissues derived from NSG mice after a 5-month transduction period. In the discussion section, we underscored the profound implications of tr-HUCMSC-mediated gene therapy in hemophilia B. We further analyzed the results and elucidated the underlying mechanisms within the discussion section. tr-HUCMSC-mediated gene therapy may exhibit advantages over traditional gene therapy, particularly in dosage requirements, organ exposure, and cost-effectiveness. Furthermore, we demonstrated the efficacy of tr-HUCMSC-mediated gene therapy in the serum of a hemophilia B patient. Additionally, we clarified the potential reasons for the in vivo secretion of hFIXco by tr-HUCMSCs. We also delved into the pertinent issue of elucidating the potential mechanism responsible for the absence of immune response in F9-KO mice during the experimental process (please see detail in the results and discussion section).

3. Conclusion: not properly written.

Answer: Thanks for your suggestion. We have rewritten the conclusions as: We have discovered a novel and safer HUCMSCs mediated approach potentially effective for gene therapy in hemophilia B.

4. Results and conclusion: The section devoted to the explanation of the results suffers from the same problems revealed so far. Your storyline in the results section (and conclusion) is hard to follow.Moreover, the conclusions reached are really far from what one can infer from the empirical results.

Answer: Thanks for your suggestion. We have rewritten many sentences in the result section and conclusion part. In results part, we highlighted the presence of healthy life during the whole experiment and non-clonal expansions of transduced cells in tissues derived from NSG mice after a 5-month transduction period. In the discussion section, we underscored the profound implications of tr-HUCMSC-mediated gene therapy in hemophilia B. We further analyzed the results and elucidated the underlying mechanisms within the discussion section. tr-HUCMSC-mediated gene therapy may exhibit advantages over traditional gene therapy, particularly in dosage requirements, organ exposure, and cost-effectiveness. Furthermore, we demonstrated the efficacy of tr-HUCMSC-mediated gene therapy in the serum of a hemophilia B patient. Additionally, we clarified the potential reasons for the in vivo secretion of hFIXco by tr-HUCMSCs. We also delved into the pertinent issue of elucidating the potential mechanism responsible for the absence of immune response in F9-KO mice during the experimental process (please see detail in the results and discussion section).

5. The discussion should be rather organized around arguments avoiding simply describing details without providing much meaning. A real discussion should also link the findings of the study to theory and/or literature.

Answer: Thank you for your suggestions. We have rewritten many sentences in the relevant points, and reorganized the discussion. We deleted many details in experiments useless in the discussion part, analyzed the results and explained the possible mechanism behind them by citing relevant literature (please see details in the discussion section) .
6. Spacing, punctuation marks, grammar, and spelling errors should be reviewed thoroughly. I found so many typos throughout the manuscript.

Answer: Thank you for your careful review. We have checked very carefully for entire manuscript and changed accordingly.
7. English is modest. Therefore, the authors need to improve their writing style. In addition, the whole manuscript needs to be checked by native English speakers.
Answer: Thank you very much for your suggestions. We have asked native speaker to edit our manuscript.


This is the end of our answering letter. If you have any further questions, please do not hesitate to contact us immediately. Thank you and our reviewers very much again for their careful comments and suggestions.

Sincerely yours,

Yongmin Tang, MD, Zibin Bu, MD, PhD
